# In Vitro Innovation of Tendon Tissue Engineering Strategies

**DOI:** 10.3390/ijms21186726

**Published:** 2020-09-14

**Authors:** Maria Rita Citeroni, Maria Camilla Ciardulli, Valentina Russo, Giovanna Della Porta, Annunziata Mauro, Mohammad El Khatib, Miriam Di Mattia, Devis Galesso, Carlo Barbera, Nicholas R. Forsyth, Nicola Maffulli, Barbara Barboni

**Affiliations:** 1 Unit of Basic and Applied Biosciences, Faculty of Bioscience and Agro-Food and Environmental Technology, University of Teramo, 64100 Teramo, Italy; vrusso@unite.it (V.R.); amauro@unite.it (A.M.); melkhatib@unite.it (M.E.K.); mdimattia@unite.it (M.D.M.); bbarboni@unite.it (B.B.); 2 Department of Medicine, Surgery and Dentistry, University of Salerno, Via S. Allende, 84081 Baronissi (SA), Italy; mciardulli@unisa.it (M.C.C.); gdellaporta@unisa.it (G.D.P.); n.maffulli@qmul.ac.uk (N.M.); 3 Interdepartment Centre BIONAM, Università di Salerno, via Giovanni Paolo I, 84084 Fisciano (SA), Italy; 4 Fidia Farmaceutici S.p.A., via Ponte della Fabbrica 3/A, 35031 Abano Terme (PD), Italy; DGalesso@fidiapharma.it (D.G.); CBarbera@fidiapharma.it (C.B.); 5 Guy Hilton Research Centre, School of Pharmacy and Bioengineering, Keele University, Thornburrow Drive, Stoke on Trent ST4 7QB, UK; n.r.forsyth@keele.ac.uk; 6 Department of Musculoskeletal Disorders, Faculty of Medicine and Surgery, University of Salerno, Via San Leonardo 1, 84131 Salerno, Italy; 7 Centre for Sports and Exercise Medicine, Barts and The London School of Medicine and Dentistry, Mile End Hospital, Queen Mary University of London, 275 Bancroft Road, London E1 4DG, UK; 8 School of Pharmacy and Bioengineering, Keele University School of Medicine, Thornburrow Drive, Stoke on Trent ST5 5BG, UK

**Keywords:** in vitro, tendon differentiation, stem cells

## Abstract

Tendinopathy is the term used to refer to tendon disorders. Spontaneous adult tendon healing results in scar tissue formation and fibrosis with suboptimal biomechanical properties, often resulting in poor and painful mobility. The biomechanical properties of the tissue are negatively affected. Adult tendons have a limited natural healing capacity, and often respond poorly to current treatments that frequently are focused on exercise, drug delivery, and surgical procedures. Therefore, it is of great importance to identify key molecular and cellular processes involved in the progression of tendinopathies to develop effective therapeutic strategies and drive the tissue toward regeneration. To treat tendon diseases and support tendon regeneration, cell-based therapy as well as tissue engineering approaches are considered options, though none can yet be considered conclusive in their reproduction of a safe and successful long-term solution for full microarchitecture and biomechanical tissue recovery. In vitro differentiation techniques are not yet fully validated. This review aims to compare different available tendon in vitro differentiation strategies to clarify the state of art regarding the differentiation process.

## 1. Introduction

Tendons connect muscles to bones and allow movements. Lesions and inflammation can occur in tendons because of mechanical stress, aging, genetic predisposition, or lesions and inflammation [1]. Tendinopathy is term used with refering to tendon disorders, and it is a generic descriptive term for the clinical condition in and around tendons arising from overuse [1]. The major conditions affecting tendons are tendinitis and tendinosis; the first is characterized by inflammation and pain, while the second is probably caused by tendinous degeneration [2]. In tendinopathy, the homeostasis of the tissue may be deeply affected resulting in permanent changes of the native tendon structures and mechanics [3]. Spontaneous adult tendon healing results in scar tissue formation and fibrosis, and it is accompanied by alterations in the biomechanical properties of the tissue. Adult tendons have a limited natural healing capacity and often respond poorly to current treatments focused on exercise, drug delivery, and surgical procedures [3]. The incapacity of complete healing derives from the nature of tendon with its poor cellularity, limited vascularization, and low metabolism [3,4]. The cellular component of the tendon is very low, and with age, it tends to diminish and change in morphology, with loss of stemness markers [5,6,7]. As the tendon is a mechanosensitive tissue and extracellular matrix (ECM) remodeling is influenced by mechanical stimulation [8,9], prolonged rehabilitation is considered a valid alternative to surgery that offers great support and is more efficient than pharmacological therapy. Regeneration, studied in fetal healing, is characterized by restoration of the native structural and functional properties of the tissue, without scar formation [10]. Therefore, it is of great importance to identify key molecular and cellular processes involved in the progression of tendinopathies and in tendon response to them to develop effective therapeutic strategies and drive the tissue toward regeneration. Unfortunately, the understanding of tendon biology and healing is incomplete, and the development of innovative treatment modalities is still lagging behind increasing demands [1]. To treat tendon diseases and support tendon regeneration, cell-based therapy, as well as tissue engineering approaches, are now considered as potential approaches to reproduce a safe and successful long-term solution for the full microarchitecture and biomechanical tissue recovery. The first step prior to any stem cell-based procedure or tissue-engineered approach to improve tendon healing and regeneration in vivo is the in vitro model. In vitro techniques are fundamental to:

- Identify and/or compare the tenogenic plasticity of different stem/progenitor cell sources,
- Define and drive cell mechanism and environmental conditions leading tenogenesis,
- Control step-wise signaling molecules and pathways,
- Direct stem cell pre-commitment before transplantation (reducing tumorigenic risks with embryonic stem cells (ESCs), unwilling differentiation path of mesenchymal stem cells (MSCs) or to increase tissue integration),
- Study the tenogenic properties of stem cells,
- Test teno-inductive properties of new scaffolds,
- Validate biomechanical teno-inductive stimuli.

**Figure 1:**
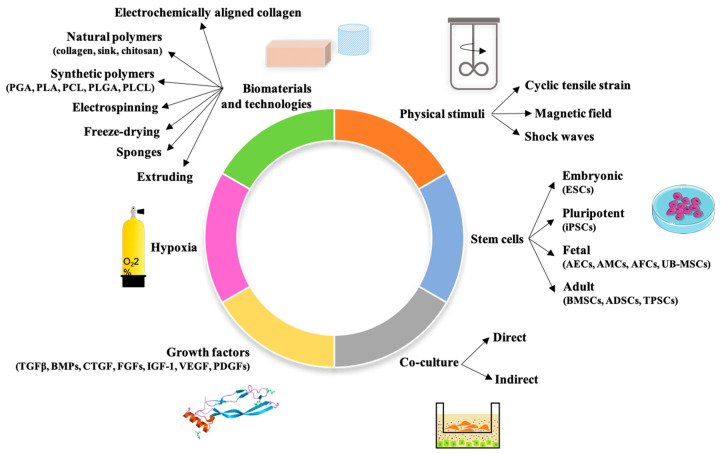
In vitro strategies for tendon tissue engineering. Tendon tissue engineering refers to a multidisciplinary field that aims at the inducement of tissue repair or regeneration. Therefore, it involves the combination of several key factors, such as cells, scaffolds, biochemical and mechanical inputs to produce a functional tendon-like construct. Abbreviations. PGA: polyglycolic acids; PLA: polylactic acids, PCL: polycaprolactones; PLGA: poly(lactic-co-glycolic) acids; PLCL: poly (lactil-co-captolactone) acids; ESCs: embryonic stem cells; iPSCs: induced pluripotent stem cells; AECs: amniotic epithelial stem cells; AMCs: amniotic mesenchymal stem cells; AFCs: amniotic fluid stem cells; UB-MSCs: umbilical cord mesenchymal stem cells; BMSCs: bone marrow mesenchymal stem cells; ADSCs: adipose derived mesenchymal stem cells; TPSCs: tendon progenitors stem cells; TGFβ: transforming growth factor beta; BMPs: bone morphogenetic proteins; CTGF: connective tissue growth factor; FGFs: fibroblastic growth factors; IGF-1: VEGF: vascular endothelial growth factor; PDGFs: platelet-derived growth factor.

At this current time, in vitro differentiation techniques are not yet validated. This review aims to compare available in vitro tendon differentiation strategies to clarify the state of art with respect to a differentiation process that remains an open biological challenge for researchers, bioengineers, and clinicians. We will begin with tendon structure, functions, and biology before describing different sources of stem cells, and finally proceeding with collating the most used tendon-inductive techniques (hypoxia, physical stimuli, biomaterials, growth factor, and co-culture) (Figure 1).

### 1.1. Tendon Structure

Tendons are fibro-elastic structures that connect muscles to bones or other insertion structures, have a high resistance to mechanical loads, and allow the conduction, distribution, and modulation of the force exerted by the muscles to the structures to which they are connected. The point of union with the muscle is the myotendinous junction, whereas the point of union with the bone is the osteotendinous junction or enthesis. Healthy tendons have high strength and minimal elasticity to resist mechanical loads. Human tendons rupture happens at 8% strain, while 4% strain produces plastic deformation. Tendons are made up of millions of a “base unit”, the fascicle, which consist of twisted bundles of collagen fibrils, whose number and thickness determines the final size of the tendon [11].

#### 1.1.1. Paratenon, Epitenon, and Endotenon

Tendons are surrounded by a loose areolar connective tissue called paratenon, whose main components are type I and type III collagen fibrils, elastic fibrils, and synovial cells lining the inner surface of the paratenon. It is elastic, and allows free movement of the tendon with respect to the surrounding tissues.

The epitenon is a thin connective tissue sheath that surrounds the entire tendon below the paratenon that contains the vascular, lymphatic, and nervous structures. The epitenon and paratenon together are often defined as the peritendon.

The epitenon on its inner surface is in contact with the endotenon, which wraps around the collagen fibers themselves and contains the blood vessels, nerves, and lymphatics [12].

#### 1.1.2. Tendon Properties

**Figure 2:**
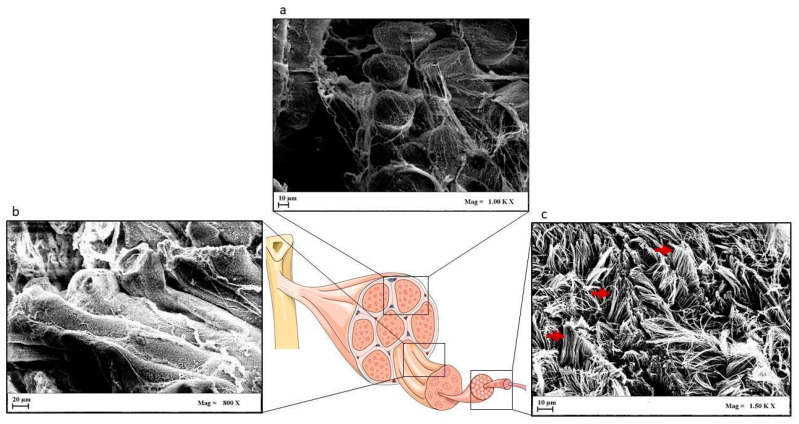
Hierarchical arrangement of the structure of tendons: (**a**) Scanning electron microscopy (SEM) of a transverse section of collagen fiber bundles (scale bar = 10 μm); (**b**) SEM image of longitudinal collagen bundles in which their parallel arrangement along the longitudinal axis of the tendon is clearly shown; each collagen bundle is surrounded by the endotenon (scale bar = 20 μm); (**c**) SEM image that shows the multiple collagen fiber bundles that make up the tendon. The sample has been cross-sectioned, but it is clearly evident the parallel orientation of the collagen fibers (red arrows) (scale bar = 10 μm). Tendon images were obtained by field emission-scanning electron microscopy (FE-SEM, mod. LEO 1525; Carl Zeiss, Oberkochen, Germany). Samples were fixed in 4% paraformaldehyde (PFA), dehydrated with critical point dryer (mod. K850 Emitech, Assing, Rome, Italy), and cut before to be coated with a gold (250 Å thickness) using a sputter coater (mod.108 Å; Agar Scientific, Stansted, UK), courtesy of Giovanna Della Porta and Electron Microscopy Labs at Dept. of Industrial Engineering, University of Salerno.

The structure of tendons is organized to provide resistance against longitudinal stresses generated by muscles. Microscopically, healthy tendons are dense connective tissues predominantly composed of parallel, closely packed collagen fibers and cells within a well-ordered extracellular matrix (ECM). Collagen represents the major component (60% to 85% dry weight) of the ECM, collagen type I being the most abundant and responsible for the fibrous structure [12]. In the tendon, collagen molecules are arranged in a hierarchical manner. Collagen is alternated with the ground substance, a less fibrous and high hydrated matrix (Figure 2) [13]. This organization is called fiber composites, and collagen is structured in an aligned fiber composite in each level of hierarchical levels from nano- to macro-scale. Collagen type I molecules aggregate to form collagen fibrils, the basic nanostructural tendon unit. In particular, the soluble form of tropocollagen molecules crosslinks in order to produce insoluble collagen molecules that gradually aggregate into defined units, which are clearly visible under the electron microscope and referred to as collagen fibrils. Bundles of fibrils form fibers, which become fiber groups, into fiber bundles or fascicles. Collagen fascicles are aligned in the direction of force application [14]. Each fascicle is surrounded by a connective tissue compartment (endotenon) to form larger bundles that are surrounded by another connective tissue sheath (epitenon), where nerves, blood vessels, and tendon stem/progenitor cells are situated.

The collagen fibers are spatially organized in a different manner according to the various structures that make up a tendon: those in the epitenon have a mainly longitudinal path, while in the peritenon, they become oblique and transverse, and finally, in the endotenon, they have a complex three-dimensional structure [15].

The complexity of a tendon structure is very important where its basic function is to transmit the force created by the muscle to the bone to make joint movement possible. This is determined by the macro and complex microstructure of tendons and tendon fibers. During the various stages of the movement, tendons are exposed to longitudinal, transverse, and rotational forces. In addition, they must be able to withstand the contusion and compression. The three-dimensional internal structure of the fibers forms a buffer system against the forces of various directions and thus prevents damage and breakage of the fibers. The alteration of the physical forces that influence a tendon in increasing or reducing stress or compressive load causes a marked and predictable change in the tendon composition and structure. In general, stress segments may have greater responsiveness and regeneration than pressure areas. This may partly explain the clinical problems encountered in the treatment of tendon injuries within the tendon sheath.

Tendon cells, which are localized in the tendons ECM, are key players in growth, maintenance, ECM synthesis and turnover, homeostasis, and remodeling in the case of minor or more severe disturbances to tissue. Mature tendons contain predominantly tenocytes and tenoblasts, which account for around 90–95% of the cell population [3].

Since there is no specific marker for these cells, the terms simply refer to cells of different shapes [16]. Tenocytes are spindle-shaped, fibroblast-like cells with elongated nuclei and a thin cytoplasm that form a complex network of cytoplasmic processes that link adjacent cells via gap junctions [5,17]. Gap junction communication is essential to create networks amongst tenocytes that can exchange ions and small molecules (<1 kDa), ensuring electrical coupling [18] and facilitating the diffusion of signaling and nutrients in this poorly vascularized tissue [19]. Gap junction communication in tendons allows the coordination of synthetic responses to mechanical stimuli (i.e., mechanotransduction). Tenocytes are terminally differentiated cells typically anchored to the collagen and located throughout tendon tissue. Fully differentiated tendon cells (tenocytes) are localized between the collagen fibers [1]. The resident tenocytes finely regulate the anabolic and catabolic processes taking place in the extracellular matrix, and they mediate tendon repair by a complex modulation of tendon homeostasis. Recently, specific miRNAs have been described for tendon matrix healing and regeneration [20].

Tenoblasts are relatively round cells with larger ovoid nuclei contained mainly in the endotenon [16]. They are immature tendon cells that give rise to tenocytes. It is suggested that tenoblasts are dominant in young tendons and that they transform into tenocytes during maturation and aging [21]. Resident tendon stem/progenitor cells (TSPC) have been recently characterized in tendon tissue of several species [22,23,24,25]. TSPCs represent 1–4% of tendon resident cells, and they exhibit the same characteristics as adult mesenchymal stem cells (MSC) [25]. TSCPs can be sorted on CD44 positivity [26] and express MSC markers Stro 1 and CD146 and tenogenic markers α-smooth muscle actin (α-Sma) and tenomodulin (Tnmd) [21,27,28]. The characteristics of this cell population are affected with age; in particular, their number and self-renewal potential decrease with time [26]. This could explain the low ability of adult tendons to spontaneous healing.

#### 1.1.3. Tendon Components

Tendons are a fibrous connective tissue formed mainly by collagen fibers, which determine mechanical and physiological properties, and elastin fibers that give it elasticity [29]. Collagen and elastin are immersed in a matrix of proteoglycans and water, where the collagen is 60% to 85% of the dry mass of the tendon, while the elastin is just 2% [30]. Collagen type I is the predominant protein, with small amounts (about 5%) of collagen type III and type V.

(1) Collagen

The basic unit of collagen type I is formed from tropocollagen, which is a protein made by three polypeptide chains that give rise to a right-handed triple helix. The alpha chains of collagen are characterized by a specific repeating triplet of amino acids: glycine, proline and 4-hydroxyproline. The glycine residue, every three positions, allows the spiraling of the chains, while the other two amino acids stabilize the helix through the formations of hydrogen bonds. The presence of hydroxylysine is also essential for the formation of intermolecular cross-links, which are responsible for the high tensile strength of collagen fibers [31]. The hydroxylation of proline and lysine residues, having fundamental importance for the stabilization of the tendon structure, takes place through specific enzymes (hydroxylase) utilizing as a cofactor vitamin C, which is an essential micronutrient for the health of the tendon.

Collagen degradation is provided by collagenases that cut the alpha chain collagen, leading to despiralization and denaturation of the molecules that occur through cathepsin G, chymotrypsin-like, and elastase proteolytic enzymes [32].

Collagen type III is the second most abundant matrix collagen protein component, and it regulates the size of type I collagen fibrils during collagen fibrillogenesis [32]. It could be found also in the endotenon [33], but its function is still unknown.

In the center of collagen I fibrils, there is collagen type V, which is probably involved in fibrillogenesis [34]. Low amounts of non-fibrillar collagens are found in tendons such as collagen type VI in the pericellular matrix. Collagens types XII and XIV provide a connection between type I collagen and other matrix molecules, and they have a role in the tendon development process [35].

(2) Elastin

Elastin, the most abundant and core protein of elastic fibers, is an essential structural constituent of tendons responsible for maintaining structural integrity and elasticity during normal function, allowing the tissue to return to its original shape once subjected to tensile force or strain. In some tendons, elastin acts to increase the efficiency of locomotion by stretching and storing energy during landing, which it can release later during the locomotion cycle [36]. In addition, it allows the return of the tissue to its original shape without energy input, which is extremely important during regeneration. Elastin is composed of amino acids glycine, valine, alanine, and proline, which form the basic units of tropoelastin, and they are joined together by covalent bonds to give a strong and elastic structure [29]. Elastogenesis is the process responsible for the formation of elastin within the elastic fibers. However, even if elastin is considered one of the most stable proteins in the body due to its highly resistance to proteolytic degradation, its turnover is limited to the physiological reduced postnatal elastogenesis, making the regeneration of the elastic fibers extremely compromising [37]. Moreover, with aging, elastin content is reduced, which may contribute to increasing tendon stiffness and reduce its resilience, leading to a complete tendon regeneration failure [38].

(3) Proteoglycans

The most abundant tendon non-fibrous protein is proteoglycans, and they are the 1–5% of the tendon dry weight. They have a core protein attached to one or more polysaccharides called glycosaminoglycan (GAG), which are negatively charged and attract water into the tendon [13]. Decorin is the most abundant proteoglycan and represents 80% of the total proteoglycan. Decorin has an important role in fibrillogenesis during development and maturation. Together with biglycan and lumincan, decorin is involved in the early stage of development [39]. Decorin also influences the mechanical properties of tendons, transferring the load to collagen fibrils and promoting slides between fibrils [13,40]. Tendons, as well as cartilage and bone, are also rich in other GAGs such as chondroitin sulfate [41].

(4) Glycoprotein

Glycoproteins are glycosylated proteins that have a similar structure to proteoglycans with less branched components. These structural proteins create a “bridge” between the molecules present in the ECM and the cell component present in the same matrix [4,29]. The most represented glycoprotein in tendons is cartilage oligomeric protein (COMP). It is located in the inter-fibrillar matrix, but it is not present in the endotenon [33]. COMP’s role is uncertain; as knockout mice did not show any tendon defect [42]. Another glycoprotein present in tendons in low quantity is Tenascin-C (Tnc), which is composed of six subunits bound together by *N*-terminal inter-chain cross-links. It may have a role in tendon elasticity, as it is present in the tendon region submitted to high forces, and its levels are modulated by mechanical stress [43].

#### 1.1.4. Tendon Vasculature

Tendons are vascularized, but less than muscles and ligaments, and the level of vascularization depends on the structure and site. Nutrients can also reach tendons due to the diffusion of synovial fluid that provides a significant amount of nutrients for many tendons [44]. The tissues enclosing and surrounding the tendon provide cellular and vascular factors that are useful for healing and the nutrition of the internal tissue. During development, tendons are supplied with a rich capillary network and have high cellular and metabolic activity [5,45]. However, mature tendons are poorly vascularized [46]. Blood vessels are generally arranged longitudinally within the tendon, passing around the collagen fiber bundles in the endotenon [5,47].

Angiogenesis is regulated by a series of growth factors and cytokines whose role is not yet well identified in normal, injured, and healing tendons [48,49]. Vascular endothelial growth factor (VEGF) has the key role in tendon healing, as it is expressed in early stages of the healing process [48]. VEGF is also a key element of homeostasis restoration during regeneration, and it contributes to the ECM biomechanical properties [50]. Active angiogenesis is required for the formation of the intravascular plexus after injury during the formation of granulation tissue, but for the complete recovery of the biomechanical properties, a specific blood vessel network must be formed to replace the vascular plexus [51].

In this context, it has been demonstrated with stem-cell based therapies conducted on experimental injured tendons that amniotic epithelial stem cells (AECs) induce healing through a rapid blood vessel regression and remodeling that promotes a regenerative program [50,52]. Even if the angiogenetic mechanisms involved in tendon healing remain unknown, they can be related to the modulatory influence of AECs on VEGF expression/synthesis and/or to the widespread anti-inflammatory effects promoted in situ. A prompt VEGF inhibition represents a positive event to maintain/reach tendon homeostasis [50,52].

VEGF gene therapy was applied to improve tendon vascularization [53,54]. VEGF165-transfected bone marrow-derived MSCs significantly promoted angiogenesis during graft remodeling of anterior cruciate ligament reconstruction, allowing restoration of the mechanical properties in rabbits [55].

VEGF is also demonstrated to have a role in sustaining the protection and survival of tendon cells [56]. Healthy human tenocytes express several VEGF isoforms [56], and VEGFA mRNA is strongly upregulated following hypoxia under both low- and high-serum conditions. Additionally, VEGF protein released in culture medium increased fourfold by anoxia, exercising a rescue role from cell death [56].

There is a paucity of literature concerning the distribution of receptors for potential angiogenic growth factors, such as those for VEGF (VEGFR-1, VEGFR-2, and VEGFR-3). An understanding about these growth factors and their receptors would help to further determine the role that they play in tendon injury and pathology.

#### 1.1.5. Tendon Innervation

Tendon innervation regards the surrounding structures that comprehend paratenon, endotenon, and epitenon. The tendon proper has non-innervation. Nerves in tendons are characterized by a low degree of myelinated nerves fast transmitting Aα- and Aβ-fibers and by a higher degree of unmyelinated, slow transmitting Aγ-, Aδ-, B- and C-fibers [57]. The nerves that end with Aα- and Aβ-fibers are nerve types I–III and mediate mechanoreception, while nerves that end Aγ-, Aδ-, and C-fibers are type IVa, and they are called nociceptors. Nociceptors mediate deep tissue pain and hyperalgesia, which is responsible for the pain in tendinopathy. The autonomic nerves ending with B-fibers are made of type IVb fibers, and they are mainly localized in the walls of small arteries, arterioles, capillaries, and post-capillary veins exerting vasomotor actions [58]. The peripheral nervous system is involved in the regulation of various efferent physiological responses.

Indeed, the nervous system plays an important part in pain regulation, inflammation, and tendon homeostasis. This neuronal regulation in healthy and damaged tendons is mediated by three major groups of molecules, including opioids, neuroregulators, autonomous, and excitatory glutamatergic neurotransmitters, which act on cell proliferation, the expression of cytokines and growth factors, inflammation, immune responses, and hormone release [58].

After tendon injury and during the healing phase, there is an extensive nerve in-growth in the tendon proper, followed by a time-dependent different neuronal release that is autonomous and glutamatergic, which amplifies and regulates inflammation and tendon regeneration [59].

In particular, substance P (SP) and calcitonin gene-related peptide (CGRP), are supposed to be stimulators of cell proliferation and stem cells recruitment because of their presence in sprouting free nerve in fibroblast during tendon healing [60,61]. They also stimulate the proliferation of endothelial cells [62,63], suggesting a role in angiogenesis [58].

However, if in damaged tendons there is an excessive and prolonged presence of sensory and glutamatergic neurotransmitters, this condition suggests an association with inflammatory and hypertrophic responses of the tissue, followed by an abnormal augmentation of sprouting sensory nerves and SP expression supposed to trigger pain signaling and hyper-proliferative/degenerative events associated with tendinopathy [64]. In the future, pharmacotherapy and tissue engineering strategies selective for neuronal mediators and their receptors could be used as effective therapies for tendon disorders [65].

### 1.2. Tendon Function

The tendon’s function is to transmit force from muscle and absorb external forces, limiting the damage to them [66]. They exhibit high mechanical strength, good flexibility, and an optimal level of elasticity in playing their unique role. Tendons are viscous elastic tissues that show stress, relaxation, and slow movement [67].

The mechanical behavior of collagen depends on the number and types of intramolecular and intermolecular bonds [68]. At rest, the collagen fibers and fibrils show a curled configuration [69]. The initial concave portion of the curve (“start”), where the tendon is brought to a 2% level of stress, is the lowering of the curled model. Beyond this point, the tendons are deformed into a linear style as a result of an intramolecular sliding of collagen triple helices, and the fibers become more parallel. With a mechanical stress below 4%, the tendon behaves as an elastic, returning to its original length. When stress is above 4%, microscopic damage can occur. Micro stress damage ranging from 8% to 10% occurs intra-fibril with a molecular slip [70]. X-ray diffraction studies show that the elongation of collagen fibrils initially occur as a result of molecular elongation, but the space between the molecules increases with the stress augmentation, resulting in slippage of the adjacent lateral molecules [71]. After this, the full damage occurs rapidly, and the fibers quickly entangle on themselves.

The breaking strength of tendons is related to their thickness and collagen content. A tendon with an area of 1 cm^2^ can support a weight that varies from 500 to 1000 kg. During activities such as jumping and lifting weights, tendons are intensely stressed [72]. It was measured that the Achilles tendon was submitted to a force corresponding to 12.5 times the body weight during a race [73].

The primary function of the tendon is to transfer force generated by muscle contraction to the skeleton, facilitating movement around joints and positioning the limbs, playing an important role in locomotion [74,75]. For efficient function, tendons must be strong and stiff under uniaxial tension, but also they have to retain viscoelasticity properties [76,77]. Tendon composition and the hierarchical organization of structural molecules in the ECM confer these properties to the tissue.

The correct orientation of collagen molecules within the fibrils is responsible for the high mechanical strength of the tendon. The fibrils are stabilized by chemical cross-links between collagen molecules [78,79,80]. These cross-links are formed by lysyl-oxidases that exert their enzymatic activity on specific lysine and hydroxylysine residues at the ends of the collagen molecules in the telopeptide regions, increasing the mechanical strength of the collagen fibrils [81]. At first, reducible cross-links connect two amino acids [80]. As the tissue ages, these combine with another adjacent amino acid to form mature trifunctional cross-links. The best-characterized mature cross-links are hydroxylysylpyridinoline. The amount of hydroxylysylpyridinoline in a given connective tissue is related to its mechanical function [79]; tendons have a high hydroxylysylpyridinoline content compared with other soft tissues [82], although there are substantial differences between the different types of tendons [34]. The hydroxylysylpyridinoline content does not change significantly after skeletal maturity, and these cross-links probably do not contribute to the altered physical properties of ageing tendons [83].

Some tendons are energy-storing structures, since they have the supplementary role of managing energy expenditure in humans and animals [74,84]. Compared to positional tendons (i.e., equine common digital extensor tendon (CDET) and human anterior tibialis tendon), energy-storing tendons (i.e., equine superficial digital flexor tendon (SDFT) and human Achilles tendon) are more elastic and extensible. Indeed, being subjected to high strains, they are required to stretch and recoil. Maximum in vivo failure strain for the CDET is 2.5% [85], which is almost 4 times lower than the 9.7% estimated in vitro for the SDFT [86]. By contrast, in vivo strain of 16%, recorded in the SDFT during gallop [87], is similar to the failure strain of 15–17% measured in vitro [77,88]. However, despite the SDFT having a very low safety margin, some horses compete in very top-level races without ever suffering from a tendon injury.

The calf muscles act on the Achilles tendon during contraction, determining the plantar flexion of the foot. Standing on the toes, walking, running, and jumping all depend on this contraction. The Achilles tendon sustains the person’s entire body weight during each step, so it may be subjected to substantial forces connected to speed, stride, terrain, and additional weight being carried or pushed [88]. To various degrees, tendons store elastic energy, and the Achilles tendon has been shown to be specialized in this very important capability. It has been demonstrated that the shorter the time between the switch from dorsi- to plantarflexion, the greater the elongation of the tendon. Furthermore, the higher the switch frequency, the greater the increase of the work loaded onto the tendon [89]. Dorsiflexion of the foot, immediately followed by plantarflexion (i.e., in walking, running and jumping), has been shown to store and release more elastic energy to the tendon, compared to the solely plantarflexion, which is probably because of the nearly isometric work of the muscle fibers of the calf during the switch [90]. This demonstrates that the coordination of structural elements in the muscle and tendon is important to withstand the very rapid force shifts present in these tissues. Thorpe et al. [14] reported that energy-storing tendons exhibit a specialized endotenon with low stiffness and fatigue resistance, which may enhance tendon elastic behavior in order to stretch and recoil efficiently. With aging, endotenon loses these characteristics, especially in energy-storing tendons, making them more prone to injury [91].

This could be also due to the fact that elastin, which localizes in the endotenon of energy-storing tendons, reduces in quantity and becomes more disorganized with aging [38].

Furthermore, tendon is a viscoelastic tissue, meaning it combines viscous and elastic behaviors. Tendon viscoelastic behavior [70,92] depends on age and activity, and it derives from a network of interactions that involves collagenous proteins, water, collagens, and proteoglycans. The unloading curve of a viscoelastic material/tissue does not proceed along the loading curve, and the material/tissue will not return to its initial shape and dimension immediately upon the removal of the applied deformation. In respect to this behavior, known as hysteresis, the area between the loading and unloading curve represents the amount of energy lost during the cycle. In viscoelastic materials, the hysteresis is considerable, and a lot of energy is lost during loading. It is generally thought that hysteresis derives from the reorganization of the multilevel fibers composite structure, with a water movement through the tissue [13].

Tendon development, homeostasis, pathology, and injury healing are driven by applied mechanical loads [93]. Mechanical forces are translated, by means of mechanotransduction processes, into biochemical signals that are able to activate and control key signaling pathways into tendon cells [94,95]. However, if on the one hand normal mechanical loads are essential for appropriate tendon development/maintenance and to induce anabolic responses in tendon cells [95], on the other, abnormal mechanical forces cause pathological conditions (such as tendinopathy), determining the cellular catabolic adaption of the tendon [1,96].

Mechanical in vitro tests on tendons involve separate clamps to grip the isolated tendon sample, ensuring it firmly. The tendon is loaded along its longitudinal axis, and the force and displacement are recorded at a constant speed until the tissue fails. Tendon mechanical response is described plotting the applied extension and the resulting force as a stress–strain curve. The stiffness of the samples is represented by the slope of the curve: for a stiffer tendon, a steeper gradient indicates greater forces to extend the sample [97].

**Figure 3:**
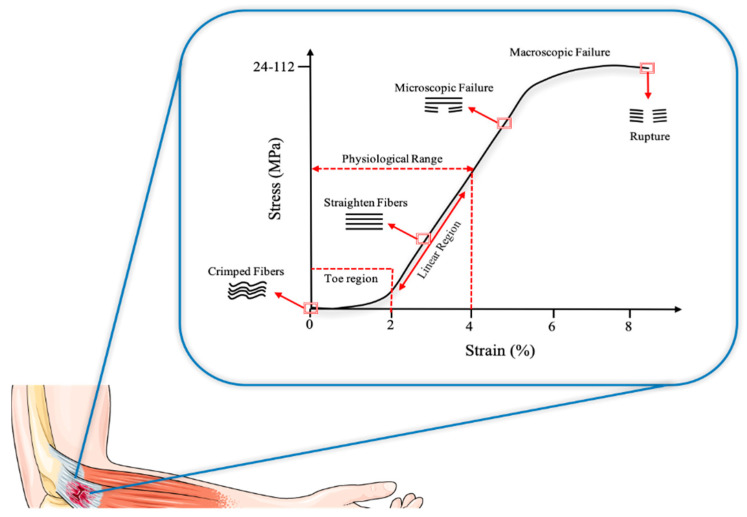
Typical stress–strain curve for tendon tissue. The schematization illustrates the behavior of collagen fibers: under tensile strain, they stretch out absorbing shock, and when the stimulus disappears, they return to their initial configuration. If the stretching limit is exceeded, overcoming the physiological range, the tissue may suffer microscopic and macroscopic traumas. Adapted from [100].

In a typical stress–strain curve, three distinctive regions can be identified (Figure 3). (1) First, there is a toe region that indicates the stretching out of the crimped pattern of the collagen fiber bundles, which is visible by polarized light microscopy. This crimped configuration, not observable under tension, acts as a buffer against fiber damage and reappears only when the stress stops and the stretched collagen bundles back to the resting state, thanks to the elastin fibers in the ECM [98]. (2) The toe region is followed by a linear region. The slope of this region is constant and represents the stiffness, or Young’s modulus. At this point, the collagen fiber bundles have no longer a crimped configuration. (3) Lastly, there is a failure region where the collagen fibers fail, leading tendon tissue to rupture. Therefore, the mechanical characteristics of the collagen fibers are directly correlated to the mechanical properties of the tendon [99]. Up to 4% elongation, a series of stretches reproduce the stress–strain curve, but when this limit is exceeded, the crimped configuration undergoes subsequent deformations not reproducing the original curve. Eight percent elongation or more, caused by acute stress, leads tendon tissue to rupture [23].

The initial concave portion of the curve (toe region), where the tendon is brought to a 2% level of stress, is the lowering of the curled model.

Recently, mechanical characterization methods have focused on an in vivo analysis of tendon mechanics, in particular about Achilles tendon mechanics. Achilles tendon mechanical properties are usually studied under an applied load. Ultrasound is used to measure the stretching, which is obtained when the muscles apply a force to the tendon [101]. However, this is feasible only for superficial tendons, focusing the in vivo studies on the Achilles or patellar tendons. The movement of the tendon-to-muscle interface is tracked by means of ultrasound within the calf, establishing tendon extension and length. Motion markers are usually arranged on a known location (the calcaneus) to check undesired movements during measurements.

This provides a considerably more representative measure of stiffness during the tendon loading range [102].

The function, age, sex, location, and species of individual tendons define their specific physiological loads. Moreover, tendon tissue is not isolated, but it communicates with both bone (enthesis) and muscle (by means of the myotendinous junction). In these transition regions, the tissue composition, material properties, and strain distributions can vary [103], often constituting the initiation sites of tendon injury, with following modifications in the cellular/matrix response [104]. The precise loading levels required for tendon repair and the exact level of stimulation (magnitude, frequency, and duration) required for tendon homeostasis remain unknown, but the comprehension of these aspects is essential to know the mechanobiological stimuli required to induce anabolic activity or reduce catabolic activity in tendon tissue [94].

### 1.3. Tendon Biology

Tendon biology is an essential key to understanding the mechanisms involved in tendon differentiation. However, little is known about tendon ontogenesis and development.

Tendon is a dynamic tissue that continually remodels itself from prenatal to postnatal life throughout adulthood and aging.

In prenatal lifetime, during embryogenesis, cartilage develops from sclerotome and muscle from myotome, while tendon ontogenesis derives from the dorsolateral sclerotome, which is called the syndetome [105]. This compartment was identified thanks to the discovery of the beta helix–loop–helix transcription factor Scleraxis (Scx) [105,106], which is an early marker of tendon development and differentiation [107,108].

Tendon specification happens firstly with the appearance of progenitor cells and secondly with the commitment and differentiation as a consequence of signaling cascade [109].

The first tendons in mice embryogenesis appear around embryonic day E9.5–12.5. These are the axial tendon progenitors that connect muscles to the spinal column. These tendons originate from the syndetome and express the beta helix–loop–helix transcription factor Scx [108].

The role of surrounding cells is crucial to drive tendons’ differentiation. In fact, the final commitment to tenocytes comes from the signal of the upper myotome that, after somite formation, expresses myogenic factor 5 (Myf5) and myoblast determination protein 1 (Myod1) in the muscle progenitors. Additionally, to controlling muscle specification, Myf5 and Myod1 are responsible for the activation of fibroblast growth factors (FGFs), whose signaling pathway induces Scx in the dorsolateral sclerotome of mouse [110].

Limb tendons develop differently when compared to the cells from which derive axial tendons. In fact, tendon progenitor cells of the limb tendons do not have a specific location in the somite, but they are localized around the lateral plate mesoderm and they are mixed with myoblasts [111,112]. FGF and transforming growth factor superfamily proteins (TGF-β) have been reported to be inducers of limb tendons [113,114,115]. TGF-β is a key signal in tenogenesis. In fact, double mutant TGFβ 2^−/−^/TGFβ 3^−/−^ mouse embryos lose tendons and ligaments in the limbs, trunk, tail, and head [116]. Recently, a new transcription factor, Mohawk (Mkx), was found to be responsible for the promotion of tendon lineage commitment and differentiation influencing the expression of collagen type I, type XIV, and tenomodulin (Tnmd) [117,118,119,120,121,122].

Tnmd is the best-known mature marker for tendons [123,124,125]. Tnmd mRNA expression was detected at day E14.5 corresponding to the differentiation stage of tendon progenitors [115]; however, Tnmd transcript was found already at embryonic day E9.5 [126]. This could mean that Tnmd is not only a mature tendon marker, but it could have a role in tendon development.

Early growth response 1 and 2 factors (Egr1/2) act as molecular sensors for mechanical signals [127] and are involved in collagen maturation and final tendon commitment [117,128,129,130]. Recent evidence has demonstrated that the protein kinase B-mammalian target of rapamycin (AKT–mTOR) axis is essential for tenogenesis. Cong et al. [131] showed its importance in mesenchymal stem cells (MSCs) and tendon differentiation. In particular, AKT–mTOR was found to be involved in collagen production and tendon differentiation, and it is a downstream signaling of TGF-β, which is involved in regulating tenogenic transcription [131].

Tendons differentiation combines the specialization of cellular compartment with the organization of the extracellular matrix (ECM), which is crucial to define tissue biomechanics properties such as elasticity and strength [132].

ECM proteins deposition is led by the transcription factors responsible for tenocytes’ development and differentiation. In particular, Scx has a crucial role in triggering the deposition of collagen type I and II. In fact, the loss of Scx in Scx^−/−^ mutant mice has a negative effect on tenocyte differentiation, resulting in the atrophy of force-transmitting tendons and into a disorganized tendon ECM [133]. Moreover, the expression of the structural collagens, Col Ia1, Col Ia2, Col III a1, and Col XIV a1, is strongly reduced as Scx controls directly collagen transcription [134,135]. In addition, the glycoprotein tenomodulin is downregulated in Scx^−/−^ mutants [133,136]. The loss of Scx also results in alteration at the structural level of tendons, disrupting the sheets surrounding the collagen fibrils [133]. This evidence demonstrates that Scx is involved tendon ECM production, which is necessary for effective force transmission.

Both Egr1 and Egr2 are fundamental for tenocyte differentiation, regulating the tendon ECM and binding to tendon-specific enhancer elements of Col Ia1 and Col Ia2, which are also bound by Scx [129,130,135]. Egr1^−/−^ mutant mice also downregulate Tnmd and these mice, in adult age, heal slowly after tendon injuries [129].

Mohawk is another regulator of ECM deposition. In fact, Mkx^−/−^ mutant mice form normal tendons at first, but later, they show reduced levels of Col Ia1, Col Ia2, Tnmd, fibromodulin (Fmod), and decorin (Dcn) as well as thinning of collagen fibrils [120,121,122]. As well as Scx, Mkx can function as a transcriptional activator after the complexation with proteins receptors of TFGβ super-family Smad 2/3 (the acronym Smad refers to the homologies of the Caenorhabditis elegans SMA and Drosophila MAD), to promote Collagen I a1, Collagen I a2, tenomodulin and decorin expression [118,119].

In postnatal lifetime, tendons retain a small population of cells with stem cell properties, which are called tendon-derived stem/progenitor cells (TSPCs) resident in the tendon stem cell niche. The stem-cell niche has been defined as a specialized microenvironment that maintains a balance of quiescence, self-renewal, and cell-fate commitment of the stem cells it hosts. The stem-cell niche is a 3D structure composed of stem cells, cytokines, and specialized ECM [137].

Bi et al. [21] showed that human and mouse TSPCs resided in a niche environment. In particular, they are localized in the long parallel chains of collagen fibrils and surrounded predominantly by ECM components. TSPCs, isolated from the stem cells niche, show adult mesenchymal stem cell (MSC) properties such as the presence of specific surface antigens, self-renewal, clonogenicity, and three-lineage differentiation capability (adipogenic, osteogenic, and chondrogenic). They also express tendon-related genes such as scleraxis and tenomodulin. In fact, TSPCS were proven able to differentiate into tenocytes in vitro [21,30]. Moreover, Bi et al. [21] demonstrated that ECM changes affect TSPCs’ fate and behavior. Of note, stem cell properties were strictly under the control of an ECM biglycan (Bgn) and fibromodulin (Fmod)-rich niche. Indeed, the depletion of two critical components using mice-deficient models resulted in impaired tendon formation and lower Scx and Tnmd expression. This is a crucial discovery in tendon biology, providing evidence of the existence of a specialized tendon stem cell microenvironment maintaining tissue homeostasis and modulating the inflammatory response, the interaction between cells, and the environment during inflammation [138]. TSPCS opened new perspectives in tendon healing and regeneration strategies even if the key aspect of the reduced availability of autologous tendon tissues continues to limit the practical impact of this discovery.

During life, ECM is continually remodeled in response to mechanical force. In fact, tenocytes actively sense mechanical stimulation, and this leads to changes in gene expression, cytoskeletal organization, and ECM protein secretion [8,9]. Remodeling of the ECM depends on the activities of matrix metalloproteinases (MMPs) and their corresponding tissue inhibitors (TIMPs), as well as disintegrin and metalloprotease with thrombospondin repeats (ADAMTS) proteases [9,139,140,141].

In an ovine model of tendon maturation from fetus to adult lifetime, the cell nuclei morphology, cellularity, PI (proliferation index), and Cxs 43 and 32 (connexins involved in gap junction present mostly in immature tendon) decreased. Moreover, biochemical changes induced a dramatic reduction of ECM molecules, growth factors, such as TGFβ1, vascular endothelial growth factor (VEGF) and nerve growth factor (NGF), as well as blood vessels and nerve fibers in adult tissues [5]. Further, molecular changes in senescent accelerated mouse induced acute tendon lesion [142]. In particular, they demonstrated increased inflammation and decreased tendon remodeling in injured aged tendons. In fact, they found an upregulation of interleukin (IL)-6 in an injured aged mouse, lower levels of tenomodulin and collagen type III, impaired expression of TIMP, and higher metalloproteinases [142]. Alteration in tenomodulin level causes inferior tendon repair process, resulting in adipocyte accumulation and fibrovascular scar formation during early tendon healing [143].

The ability of tendon-derived stem/progenitor cells to differentiate into tenocytes diminishes with age [133,144]. Aging in tendons results in morphological and molecular changes that involve both cells and ECM. In fact, in adult life, tendon becomes a specialized tissue with few cells that reduce their communication and synthetic activity [7]. With aging, TSPCs lose their stem markers, and they undergo a series of changes that affect their healing ability [6].

Taken together, these data suggest that aging affects the ability to repair after injury.

Next to physiological aging, there are a series of pathological situations that affect tendons such as injury or tendinopathy [3].

Thanks to new technologies in proteomics, we know that when tendinopathy occurs, there are changes in the expression of many ECM tendon proteins [3,145]. For example, there is an increase in collagen I and III, metalloproteinase (MMP)-1-9-13, tissue inhibitor of metalloproteinase (TIMP)-1, and VEGF with a decrease in MMP-3 [146].

Tenascin-C expression is sensible to mechanical strain and it is upregulated with tendinopathy. Glycoproteins such as fibronectin and thrombospondin have a role in tendon repair as they are highly expressed during tendon regeneration [3]. Proteoglycans enable the diffusion of water-soluble molecules and the migration of cells into areas of tendon injury. Type III collagen is overexpressed during repair, and it is replaced by type I collagen during post-injury remodeling [147]. The ratio of type III to type I collagen may be an indicator of the tendon repair process [147].

All the evidence collected to date demonstrated that tenogenesis is a stepwise process characterized by sequential markers. Scx is the early marker of tenogenesis, and it is also considered a crucial gene in adult tissue during the early phase of progenitor cell commitment [108] and in modulating tenocyte mechanotransduction. Mendias et al. [148] demonstrated that Scx-GFP mice subjected to a treadmill training program increased gene expression of scleraxis, tenomodulin, and type I collagen [148]. Mutant mice Scx^−/−^ showed alteration in tendon matrix and disorganization that led to an intermixing of tenocytes and endotenon cells. Moreover, these mice had a limited use of paws and were unable to move the tail [133].

Equine tendon fibroblasts exposed to siRNA targeting of Scx showed an impaired ability to migrate on softer surfaces, and they exhibited differences in focal adhesion morphology compared to controls, suggesting a potential role for Scx in modulating tenocyte mechanotransduction [149].

Dyment et al. [150], using a murine patellar defect model and Scx-GFP reporter mice, described an Scx role during the healing process. After injury, the cells of the paratenon that normally do not express Scx migrated toward the defect site and expressed scleraxis and smooth muscle actin alpha by day 7. Cells contained in the injured site displayed an increase of Col I and Col III but a decreased expression of tenogenic transcription factors (Scx and Mkx) and collagen assembly genes (Fmod and Dcn). By contrast, Egr1/2 and Tnc were upregulated. These results suggest that paratenon cells, which normally do not express Scx, turn on Scx as response to an injury, and they deposit matrix to overcome the defect [150]. This evidence demonstrated that Scx is not only involved in tendon development, but it has a role also in adult physiology and illness.

Mohawk can be considered another marker of tendon development as it regulates the deposition of Col I and tenascin C through the binding of Smad3. In fact, Mkx^−/−^ mutant mice show defects in total collagen deposition and a small collagen fibers diameter. Moreover, the tendon tensile strength of mutant mice is decreased, suggesting a role of Mkx in affecting the mechanical properties of tendons [120].

Early growth response 1 and 2 factors (Egr1/2) are involved in tendon differentiation, regulating transcription factors and ECM deposition. In fact, knockout mice display less Scx and Col I expression [130]. Egr also displayed functions in tendons healing as tendon-injured mice, after Egr1-transfected cells, showed an increase in tendon gene expression, including Egr1, Scx, Col I a1, Col I a2, and Tnmd [129].

Furthermore, the mammalian target of rapamycin (mTor) is emerging as an important regulator of tenogenesis. It has a role in tendon differentiation, as it was upregulated in the tenogenesis of MSC [131]. Apparently, it is involved in tendon healing, as it is downregulated in tendinopathy tissue. Moreover, an ablation of mTor in tendons results in less deposition of Col I [131].

Mature tendons are characterized by the expression of tenomodulin and thrombospondin [151].

Tenomodulin was first discovered by Brandau et al. [126] and Shukunami et al. [151], and it is considered a late marker in tendons [136]. Studies demonstrated that Tnmd is downregulated in Scx and Mkx mutant mice, suggesting that in tendon development Tnmd is regulated by these transcription factors [122,133]. Tnmd knockout mice revealed reduced tenocyte proliferation, premature ageing of TPSCs, and abnormal collagen fibrils [123,124].

In human tendon rupture, Tnmd was downregulated, while VEGF and MMP 1, 2, and 13 increased [152]. Tnmd presence has a positive role in tendon and cells function [153].

Thrombospondin (Thbs) is considered another late tendon marker [154]. It was demonstrated that knockout mice displayed abnormal collagen fibrils, glycosaminoglycan modifications, decreased expression of a TGF-β receptor beta-glycan, decreased activity of lipoprotein lipase, and decreased uptake of very low-density lipoprotein (VLDL), and forelimb grip strength was reduced [155]. Thbs has a role in regulating ECM deposition and also in the repair of myotendinous junction (MTJs) [156].

Tenascin C is an important component of the extracellular matrix, and it was found in MTJs [157,158]. Moreover, it could play a role in collagen fibers orientation [157]. Tnc is regulated by mechanic stimulation, and it is upregulated in the case of tendinopathy [158,159,160]. An interesting study of Mehr et al. [160] showed that Tcn mRNA and protein expression was upregulated, in vitro, in the portion of tendon subjected to compression with respect to the portion not directly affected by mechanical stimulation. Moreover, they demonstrated that the Tnc used in culture is able to decrease cell adhesion to fibronectin. All these data suggest that Tnc is modulated by mechanical stimuli and has a role in maintaining the fibrocartilaginous region of tendons [160].

Among tendon markers, Col I and Col III can also be considered. They are the major component of ECM, and their deposition is controlled by transcription factors Scx, Egr1/2, and Mkx [120,130,134]. Collagen content is essential for healthy tendons. In fact, alteration of the Col I and Col III ratio can be an indicator of tendinopathy [147]. Moreover, during the healing process, they have different functions, as Col III appears during the proliferative phase and Col I is more deposited during the last phase of remodeling [3].

Therefore, the transcription factors Scx, Egr1/2, Mkx, mTOR, and molecules tenomodulin, thrombospondin, tenascin C, and collagen type I and III can be considered as the most important markers associated with tendons.

Tendon biology comprehends different factors that together create a delicate balance that can be easily disrupted. Tissue engineering could be a solution to tendinopathy, because tendons are not able to repair themselves properly during aging or after injuries.

## 2. In Vitro Tenogenesis Techniques

**Figure 4:**
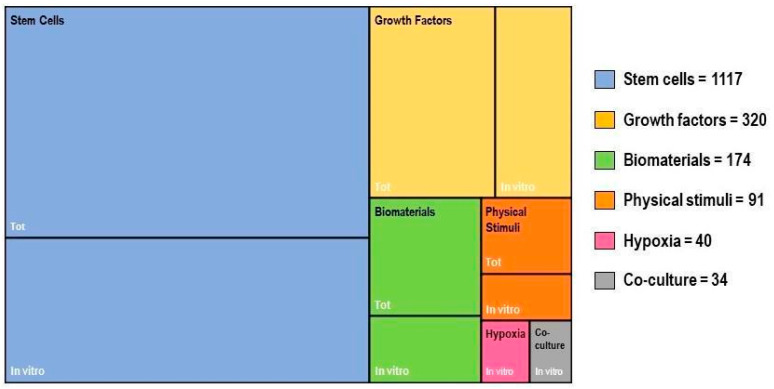
Scientometric analysis aimed to compare the available publications on the Scopus database related to the main topics on tendon differentiation discussed in this review. The legend indicates the number of total publications for each topic, whereas the figure also represents the sub-class of papers exclusively referred to the in vitro conditions.

**Figure 5:**
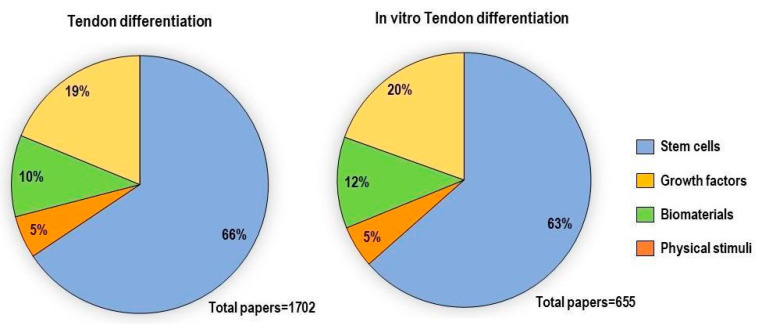
The comparative scientometric analysis of available publications on the Scopus database on tendon differentiation and in vitro tendon differentiation reveals four main common topics: stem cells, growth factors, biomaterials, and physical stimuli. The topic papers’ distributions amongst topics are independent of tendon differentiation sub-category (tendon or in vitro tendon differentiation). Stem cells are the most represented one (approximately 65%) followed by growth factors (approximately 20%), biomaterials (approximately 10%), and finally physical stimuli (for both 5%).

In vitro tenogenic techniques are fundamental to understanding tendon biology and to mimic the physiological environment that allows tenogenesis to proceed in vivo. The first step in assessing an in vitro technique is the choice of the cell source, and the literature generally describes the use of either tendon-derived stem cells (or tendon progenitor stem cells), tenocytes, stem cells from fetal or adult origin, including embryonic stem cells, amniotic-derived stem cells, and mesenchymal stem cells from different tissue origins (bone marrow, adipose tissue). Then, there is technique selection seeking to reproduce the complex microenvironment to support tendon differentiation. Scientometric research on the Scopus database revealed a significant bibliographic production of tendon differentiation techniques, but in vitro technique publications represent a minor part compared to the total papers found (Figure 4). Moreover, the scientometric research revealed four main topics in the field of tendon differentiation corresponding to stem cells, growth factors, physical stimuli, and biomaterials (Figure 5).

Here, we are going to focus on the in vitro application of these four main topics and two further promising in vitro techniques: hypoxia and co-culture. The term hypoxia term has relevance only when used in describing a reduction in oxygen tension below that which would routinely be considered as normoxic for that tissue. The physiological, normoxic, and oxygen conditions for a tendon in vivo due to its low levels of vascularization is in the range of 1–5% O_2_. Scaffolds are able to reproduce the ECM, mechanical stimulation is the condition to which the tendon is subjected in vivo, growth factors are naturally involved in tendon development and repair, and co-culture allows communication between tissue and stem cells, or between two different stem cells, producing a soluble factor that is able to stimulate tendon differentiation.

### 2.1. Stem Cells

**Figure 6:**
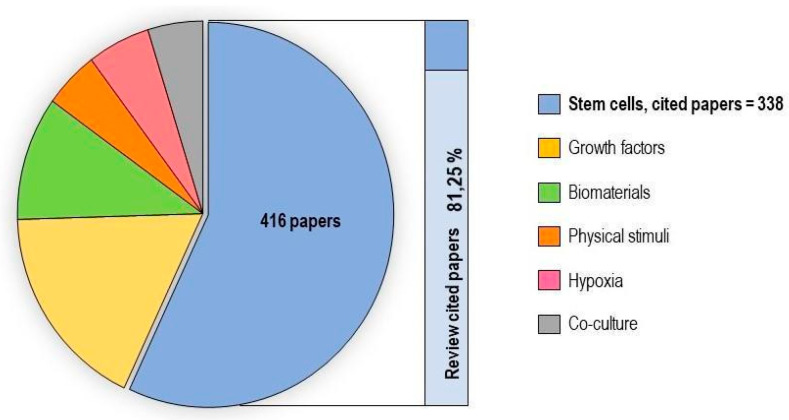
The light blue section shows the percentage/number of papers available on the Scopus database obtained by combining key words in vitro tendon differentiation with stem cells. In the histogram, the relative percentage of cited papers is expressed as well as the relative number in the legend.

Different types of stem cells sources have been explored in vitro to determine their capacity to differentiate into tenocytes for use in regenerative medicine. The scientometric analysis revealed a consistent production on this topic (Figure 6). Many techniques have been used to induce tenocyte differentiation, but a validated protocol still does not exist [161]. The validation of the tendon-inductive techniques is essential for the in vitro model, since it represents a preliminary step to all the applications in which differentiated cells can be used. The tenogenic potential of stem cells from different origins have been tested in vitro in order to find the most suitable stem cell type for applications in regenerative medicine.

Here, we will review the tenogenic potential of pluripotent stem cells, which are the most plastic, proceeding with multipotent stem cells derived from tendons and other tissue. Among pluripotent stem cells, both embryonic and induced pluripotent stem cells have been investigated. Embryonic stem cells (ESCs) could be suitable for tissue engineering because of their ability to differentiate into all tissues derived from the three germ layers [162]. The proliferation capacity of ESCs is a clear advantage by providing sufficient cell numbers [163].

The stepwise differentiation of hESCs into tenocytes through a mesenchymal transition stage has been achieved by first passaging at confluence into 10% serum replacement medium plus fibroblastic growth factor-2 (FGF-2) followed by a second passaging at confluence into 20% fetal bovine serum containing media. Then, cells were seeded at colony-forming densities and emergent colonies with fibroblast-like morphologies were designated as hESC-derived mesenchymal stem cells (hESC-MSCs) and cultured under uniaxial static tension to produce an engineered tendon. After 14 days, the construct showed a high level of Scx gene expression. The hESC-MSCs engineered tendon was also able to improve tendon healing and regeneration in vivo after implantation [164].

Chen et al. [165] demonstrated that hESCs-MSCs obtained with the above protocol [164] were induced to tendon differentiation with the combination of Scx overexpression and mechanical force stimulation. In particular, Scx overexpression led hESCs to a tenocyte commitment characterized by the expression of Col 1 and Tnc and a reduced expression of SRY-box transcription factor 9 (Sox9) [165].

Then, this protocol [164] was further elaborated when following on from the induction of mesenchymal transition, the hESCs-MSC were seeded onto a knitted silk–collagen sponge scaffold [166]. When subjected to mechanical stimulation in vitro, hESC-MSCs exhibited tenocyte-like morphology and an expression of tendon-related gene markers such as Col I, Col III, Scx, and mechanosensory structures and molecules such as cilia, integrins, and myosin. When implanted in vivo, the engineered construct resulted in enhanced tendon regeneration in situ and superior mechanical performance characteristics [166]. Mechanical stimulation free differentiation of hESCs was also proven under a 2% O_2_ condition combined with the supplementation of BMP12 and BMP13. These cells displayed a tenomodulin expression pattern and morphology consistent with that of the primary tenocyte used as control. Moreover, they demonstrated a consistent expression of Col I, Col III, Dcn, Tnc, Thsb4, and Tnmd gene levels [167].

Induced pluripotent stem cells (iPSCs) can avoid the ethical concerns associated with hESC [168,169]. In principle iPSCs should have no biological difference to hESC and should also be suitable for tenodifferentiative purposes, although to this point, few studies have explored this [170]. Recently, Komura et al. [171] have demonstrated that murine iPSCs can be differentiated into tenocytes. They created reporter mice that expressed enhanced green fluorescent protein (EGFP), driven by the promoter of the tendon-specific Scleraxis (Scx) transcription factor gene, from which they generated iPSCs. The iPSC-derived EGFP-positive cells were treated with a tenogenic differentiation protocol designed to mimic tendon development and differentiation in embryogenesis. In particular, they supplemented the culture media with growth factors and molecules involved in tendon development at different stages of culture. On day 2, Wnt3a and Activin A were added; on day 3, embryonic bodies (EBs) were cultured in differentiation medium supplemented with basic fibroblast growth factors-2 (bFGF-2). On day 5, EBs were harvested and dissociated to single cells that were cultured in differentiation medium supplemented with insulin-transferrin selenium, TGF-β1 and bFGF. These iPSCs exhibited elevated expression of tendon-specific genes, including Scx, Mkx, Tnmd, and Fibromodulin (Fmod). These cells were also able to promote tendon regeneration in mice after transplantation into injured tendons, reducing scar formation via the paracrine effect [171].

Another category of stem cells comprehends fetal and adult multipotent stem cells derived from tendons or other systems. Fetal multipotent stem cells used to reproduce tenogenesis in vitro are amnion-derived and umbilical cord stem cells. Interestingly, these cells are emerging as a new resource for tissue engineering and regenerative medicine [172,173], since they conjugate a remarkable plasticity with associated safety properties [172].

Amnion-derived stem cells can include amniotic epithelial stem cells (AECs), amniotic mesenchymal stem cells (AMCs), and amniotic fluid stem cells (AFCs). Amniotic epithelial stem cells are a relevant and promising resource for tissue engineering and regenerative medicine [174,175]. Several reports describe how AECs display anti-inflammatory [176,177,178], anti-fibroblast [179], and antimicrobial properties [180] together with a low immunogenicity and tumorigenicity [181,182]. Moreover, they can be collected from human or animal amniotic membranes from the placenta as a discarded tissue with few ethical issues [173,183].

The tenogenic potential of amniotic-derived cells has been demonstrated both in vitro and in vivo [184,185,186,187,188,189].

Barboni et al. [184] showed that ovine AECs, by following a stepwise differentiation process, can develop a fully differentiated tendon phenotype. The protocol relied on exposing AECs to a co-culture microenvironment with ovine calcaneal fetal or adult tendon explant or tenocytes that resulted in AECs displaying a tenocyte morphology and a high level expression of tendon-related genes such as Scx, Tnmd, Thsb4, Col I, and protein such as Col I and Connexine 32, 43. Moreover, they expressed mesenchymal marker αSma. Interestingly, tenocyte differentiation was optimal with AECs co-cultured with fetal tendon explant or tenocytes than with adult tendon or tenocytes.

AECs tenocyte differentiation was also tested on a poly(lactic-co-glycolil) acids (PLGA) electrospun tendon-mimetic scaffold. They displayed an expression of mesenchymal markers (Snail, Vimentin, αSma) after 48 h and tenogenic marker expression (Col I and Tnmd) after 28 days of culture [190].

AECs could be well suited to develop an understanding of the efficiency of tendon induction techniques as they lack mesenchymal and tenogenic markers when harvested but acquire them through the differentiation process [184,188,191,192].

Amniotic mesenchymal stem cells were shown to differentiate toward the tenogenic lineage in a transwell co-culture system after growth factor induction. Specifically, human mesenchymal amniotic stem cells (hAMSCs) co-cultured in a transwell system with human anterior cruciate ligament fibroblasts (hACLFs) and exposed to basic fibroblast growth factor (bFGF) and transforming growth factor beta-1 (TGFβ1) showed an increased deposition of collagen types I and III and mRNA upregulation of collagen types I and III, fibronectin, and tenascin C [193].

Moreover, amniotic fluid-derived stem cells showed the ability to differentiate into tenocytes after bone morphogenetic protein 12 (BMP-12) stimulation displaying an upregulation of Tnmd and Dcn [194].

Mesenchymal stem cells from the umbilical cord (UB) are also described as undergoing tenogenic differentiation when culture with BMP12 resulted in the expression of mohawk homeobox, collagen type I alpha 1, scleraxis, tenomodulin, and decorin at day 10 of culture [195,196].

Adult stem cells belonging to the mesenchymal stem cell family have a differentiation potential and paracrine effect reported to play a crucial role in their beneficial properties by promoting angiogenesis, stimulating local progenitor and mature cells, or regulating inflammation and immune cell functions [197]. Adult MSCs are mainly isolated from bone marrow (BMSCs) and adipose tissue (ADSC).

Bone marrow mesenchymal stem cells (BMSCs) are the most widely used stem cell type. BMSCs are described as being tenocyte differentiation competent following exposure to growth factors such as GDF5, BMP14, and/or mechanical stimulation [198,199,200].

BMSCs showed tenogenic commitment as a consequence of the combination of bone morphogenetic proteins (BMP-12 and 14) alongside transforming growth factor beta (TGF-β) and vascular endothelial growth factor (VEGF) both in 2D and 3D cultures within fibrin-based constructs. The expression of tenogenic gene markers, such as Tnc and Col I after 7 days of culture and Tnmd and Col III after 14 days [201] were noted. A fibrin hydrogel merged with an elastic braided hyaluronated band scaffold was also described as committing human BMSC into a tenogenic phenotype under cyclic strain [198,202]. Dai et al. [203] showed that BMSCs are more responsive to bone morphogenetic protein-12 (BMP-12) stimulation compared to ADSCs [204].

However, BMSCs also have some limitations, such as painful harvesting procedures with frequently low cell yield, reduced MSC quality with advanced donor age [204], ectopic ossification, and higher risk of adhesion formation when transplanted in vivo [205].

MSCs derived from adipose tissue (ADSCs) are an attractive candidate cell type due to their easy isolation, multi-potentiality, and high responsiveness to distinct environment stimuli [206]. This cell type’s tenogenic ability has been shown including when exposed to tendon extracellular matrix and TGFβ3 [207]. Moreover, ADSCs seeded on a tropoelastin-coated biomimetic scaffold, after 21 days, showed an increased protein expression of tendon-related markers such as Scx and Tnmd and were able to secrete extracellular matrix components such as Col I, Col III, Tnc, and Dcn [203].

ADSCs’ ability to undergo tenogenic differentiation was successfully tested with a tenocyte-imprinted substrate on polydimethylsiloxane (PDMS). After 14 days, ADSCs expressed tendon-related protein scleraxis and tenomodulin [208]. However, the main disadvantage of ADSCs is their preference toward adipogenesis [209].

Stem cells can also be genetically modified to either maintain a tenogenic phenotype or promote differentiation toward the tenogenic lineage [210]. A risk of cell application is a phenotypic drift of primary cells during the in vitro differentiation protocols, as ESCs and MSC can form teratoma or ectopic bone tissue, respectively. Gene transfection can be used also to improve the paracrine properties of stem cells in order to have a major effect in vivo [1].

Human embryonic stem cells transfected with Scx following on from mesenchymal transition showed tenogenic commitment after mechanical stimulation. In fact, these cells expressed more tenomodulin gene expression and more ECM deposition with respect to control cells or to those treated with only Scx overexpression or mechanical stimulation [165]. BMSCs transfected with BMP-12 induced differentiation into tenocytes enhancing Col I and Scx mRNA expression [200]. Further evidence showed that BMSC can be induced toward tenogenic differentiation after transfection with an adenoviral vector carrying bFGF or BMP-2 increasing the expression of Scx and Col I [211]. Guerquin et al. [129] demonstrated that forced Egr1 expression programmed MSCs toward the tendon lineage, promoted the formation of in vitro engineered tendons, and increased the formation of tendon-like tissues in a rat model of Achilles tendon injury. They suggested that the ability of EGR1 to promote tendon differentiation was partially mediated by TGF-β2 [129]. Hsieh et al. [212] tested Scleraxis-programmed mesenchymal stem cells (hMSC-Scx) in the healing of a rat Achilles tendon defect. hMSC-Scx reduced ectopic bone formation and increased the ECM protein expression of collagen type I and III, biglycan, decorin, lumican, and elastin [212].

Tendon-related somatic stem cells have created the possibility for tendons to use a precommited source of tissue specific stem/progenitor cells. Tendon progenitor stem cells (TSPCs) represent a particular category of multipotent stem cells being tendon-derived with inherent pro-tenogenic abilities. TSPCs were first reported and described in 2007 [21] and subsequently identified in different tendons, isolated from different species, and further characterized [213]. TSPCs express higher mRNA levels of tendon-related gene markers including the transcription factor Scx and the late differentiation factor Tnmd [214]. TSPCs spontaneously undergo tenocyte differentiation in vitro [215] and exposure to tendon ECM component in vitro, such as biglycan; they also enhance TSPCs differentiation into tenocytes, as they express late tendon-specific markers such as thrombospondin 4 and tenomodulin at gene and protein levels [93].

As TSPCs are poor in number when harvested, they need to be amplified in vitro, but their expansion leads to an overexpression of osteogenic markers and a loss of morphological characteristics [216,217]. Stem cells can be a solution to improve tendon healing and regeneration, but each of them have many advantages and disadvantages. Different techniques can be used to induce tendon differentiation, but the stepwise process could avoid unanticipated differentiation.

### 2.2. Hypoxia

Oxygen is one of the most important environmental factors for cells both in vivo and in vitro. It is a vital molecule serving as a metabolic substrate and a signaling mediator [218] in maintaining tissue homeostasis and supporting tissue regeneration [219,220]. Cellular adaptation to oxygen levels relies on a family of hypoxia-inducible transcription factors (HIFs) that sense changes in environmental oxygen and orchestrate a complex transcriptional program, especially HIF1 α, which is defined as the “master regulator” of hypoxia, as it is the best characterized key player in cellular response to hypoxia [221]. In cellular or tissue normoxic, or physiological normoxia, environments, HIF1a is constantly expressed, but its conserved proline residues are hydroxylated by Prolyl hydroxylase domain enzymes (PHDs). This oxygen-dependent hydroxylation creates a binding site for the von Hippel–Lindau (VHL) protein, which is a component of the E3 ubiquitin ligase complex that leads the HIF1α subunit to proteasomal destruction [222]. When lower than normoxic oxygen concentrations occur, the HIF-1α protein is stabilized and accumulates inside the nucleus [223], where it can induce the transcription of many genes with adaptive functions [224]. Indeed, HIFs’ contribution to oxygen homeostasis is linked to several molecular mechanisms, including the synthesis of DNA, mRNA, microRNA, and protein [225].

**Figure 7:**
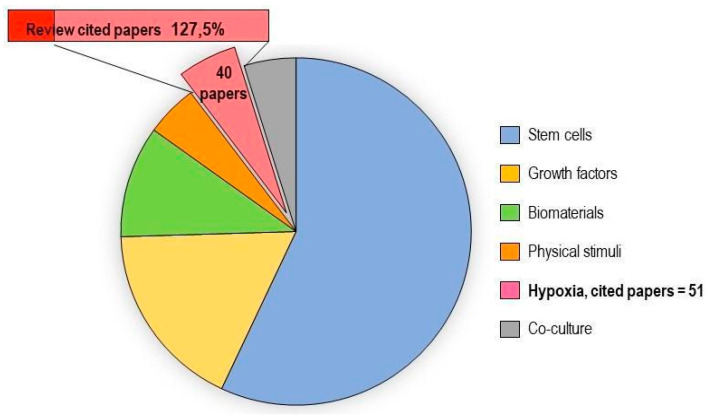
The pink section shows the percentage/number of papers available on Scopus database obtained by combining key words in vitro stem cells tendon differentiation with hypoxia. In the histogram, the relative percentage of cited papers is expressed as well as the relative number in the legend.

Curiously, while the addition of exogenous growth factors has been largely adopted in cell culture for the induction of tenogenic differentiation [226], less attention has been paid to the influence of oxygen. Oxygen plays an important role in different aspects of cell biogenesis such as metabolism, migration, angiogenesis, proliferation, differentiation, and apoptosis [227]. For this reason, low O_2_ tension cultures have been employed in recent years to reproduce physiological normoxic environments of cells [228,229]. Scientometric analysis highlights that scientific production on the use of hypoxia for in vitro tenogenic techniques remains low compared to the other techniques (Figure 7). Generally, for historical reasons, cell and tissue culture is performed at atmospheric O_2_ levels that correspond to 160 mmHg (20–21% O_2_), but once air is inspired, the oxygen pressure already begins to decrease to 150 mmHg, and when oxygen is delivered via the blood circulation into alveolus, it moves down along a gradient of about 100–120 mmHg [230]. In the body districts, oxygen tensions become progressively lower, and although it is difficult to record the exact oxygen level that cells experience within their specific microenvironment, oxygen concentrations between 2% and 9% (14.4–64.8 mmHg) have been considered as the “physiologic normoxia” [231] depending on the vascular density [219] and the balance between oxygen supply and consumption [230].

In bone marrow, a primary source for mesenchymal and hematopoietic stem cells, pO_2_ concentrations range from 1.5% O_2_ [232] to 7% [233].

Fully mineralized bone tissue exists at a very low pO_2_, and the oxygen concentration of articular chondrocytes is less than 10% at the surface and decreases at 1% in the deepest layer [234]. Hence, the blood flow regulates the tissue oxygen pressure [235], whereby less vascularized organs receive less oxygen, and their pO_2_ is significantly lowered [220].

Tendons and ligaments are poorly vascularized tissues in comparison to other body districts [75], but to the best of our knowledge, there are no exact recordings of oxygen values in tendons. Skeletal muscle oxygenation is about 2–5% O_2_ [236] ranging from 7.5 to 31 mmHg where the high variability depends on the rate of oxygen consumption of muscles [235], and the oxygen consumption of tendons and ligaments is 7.5 times lower than skeletal muscles [3]. This means that tenocytes can be anticipated to live in a physiological low O_2_ environment [237]; thus, a lower oxygen tension appears to be critical for tendon recovery and remodeling in vivo or for the preservation of resident cells phenotype ex vivo. The two major types of cells contained in tendons are tenocytes and the recently isolated tendon stem cells (TSCs) [93], which can promote tendon repair [238].

In TSCs culture, oxygen tension control promotes in vitro expansion and phenotype maintenance [239]. Primary tenocytes cultured in low O_2_ tensions (2% O_2_) showed enhanced proliferation at different passages in comparison to tenocytes cultured in air oxygen (20% O_2_), without modifications in their function and phenotype [240]. In addition, culturing tenocytes in low O_2_ tension decreased matrix metalloproteinase-1 (MMP-1) expression, leading to increased collagen deposition that could be beneficial for engineered tendon maturation [240].

Maintaining cells in vitro at low pO_2_ would also favor stemness over differentiation [241]. Indeed, under the 5% O_2_, human TSC (hTSC) grow faster, showing a significantly higher expression of stem cell marker genes levels (Oct-4 and Nanog) and exhibiting a more potent multi-differentiation capacity in terms of adipogenesis, chondrogenesis, and osteogenesis. Moreover, when implanted with an engineered tendon matrix (ETM), hTSCs cultured in hypoxic conditions produced more extensive tendon-like structures [242].

The increase of clonogenicity, cell proliferation, and DNA synthesis of hTDSC and their higher levels of tendon-related marker tenomodulin (Tnmd) at 2% O_2_ confirm that tendon normoxia might be helpful for an efficient expansion of hTDSCs in vitro and consequently for tendon tissue engineering [238]. However, a recent study reported that 5% O_2_ improved hTSCs self-renewal enabled the recovery of sufficient TSCs for tissue engineering, while the self-renewal capacity of hTSCs kept in a physiologically hypoxic 0.5% O_2_ was inhibited. Furthermore, expression levels of stem cell markers nucleostemin (NS), homeobox protein Nanog (Nanog), octamer-binding transcription factor 4 (Oct-4), and stage specific embryo antigen 4 (SSEA-4) were inhibited in 0.5% O_2_ and 20% O_2_. These results suggest that precise oxygen levels must be determined and kept within a certain range for optimal outcomes [243]. The difficulties linked to tendon repair led to the development of novel strategies for tissue replacement such as stem cell therapy, which has received increasing attention as an alternative therapeutic option [244]. In fact, oxygen tension can modulate tenogenic differentiation also in different stem cells sources, such as adipose-derived mesenchymal stem cells (ADMSCs), embryonic stem cells (ESCs), and mesenchymal stem cells (MSCs).

Evidence suggests that HIF-1a might play a role in regulating differentiation under hypoxia [224]. Indeed, HIF-1a expression was found to be significantly upregulated during adiposed derived mesenchymal stem cells (ADMSc) differentiation into tenocytes-like cells, but when ADMSCs were treated with HIF-1a inhibitor, the effect of hypoxia on the differentiation was attenuated. More in detail, the increase of collagen I and III (Col I and Col III), Tnmd, thrombospondin-4 (Thbs-4), and Scleraxis (Scx) were significantly reduced in the HIF-1a inhibitor-treated group in comparison to the vehicle-treated group in hypoxic co-culture system with tenocyte [226].

Due to the challenge of in vitro tenogenesis, it could be a good strategy to combine more factors in order to improve the development of stem cell-based therapies for tendon treatment. In this context, Dale and others [167] combined tendon normoxia and growth factors. They demonstrated that human ESCs cultures supplemented with a cocktail of bone morphogenetic protein-12, -13 (BMP-12, BMP-13) and ascorbic acid (AA) can induce tenogenic differentiation in vitro when cultured under low oxygen (2% O_2_) conditions. The stable transcription of tendon-linked and specific genes was observed alongside the deposition of a tendon-like matrix and elongated, synapsing, cells with concurrent tenomodulin expression [167]. Another factor that may influence tenogenesis is the microRNA 210 (miR-210), which has been linked to HIF1a activity [245,246]; in fact, miR-210 is upregulated in response to hypoxia. When oxygen levels decrease, HIF-1 protein and its transcriptional activity increase as well as miR-210, which triggers a positive feedback loop, suppressing the activity of a negative regulator of HIF-1, glycerol-3-phosphate dehydrogenase 1-like (GPD1L), resulting in HIF-1 stabilization [247].

The local administration of synthetic miR-210 into the injured Achilles tendon enhanced its healing via the acceleration of angiogenesis in an early phase. After 2 weeks of recovery from the surgery, they found regular dense collagen fibers with higher diameter in the miR-210 treated in comparison to the control. After 12 weeks, the miR-210 group exhibited parallel and dense fibers in repaired Achilles tendons, while wavy and loose fibers were still observed in the control group [248].

MSCs are an important source in regenerative medicine and have received great attention in the tenogenic differentiation and regeneration of functional tendons [203]. When cultured in normoxic conditions, MSCs display enhanced proliferation rates, retention of stem cell properties, inhibition of senescence, and increased differentiation ability [249,250]. The therapeutic effects of MSCs transplantation could depend on the release of paracrine factors such as growth factors and cytokines [251]. Conditioned media derived from normoxic MSCs have been applied for stimulating wound and fracture healing [252]. It has been demonstrated that normoxic MSCs increased bone repair capacity in vivo in an immunocompromised mice model of a calvarial defect [253] and Achilles tendon healing with enhanced biomechanical strength compared with air oxygen cultured MSCs [254].

Finally, amniotic epithelial cells (AEC) are another source of stem cells that are able to differentiate toward the tenogenic lineage both in vitro [184] and in vivo [52]. This was evidenced in Achilles Tendon Regeneration [52] due to their spontaneous inclination toward epithelial–mesenchymal transition (EMT) during in vitro amplification [191]. Through the EMT process, epithelial cells acquire a mesenchymal phenotype, and several studies have proved that low oxygen is an important factor in the regulation of multiple genes involved in the EMT process [255,256]. HIF-1α stimulates the transcription of Twist, which is a factor that leads EMT [257]. Although there is no evidence of an oxygen effect on AEC tenogenic induction, we can speculate that the modulation of EMT by oxygen levels might improve this process in AEC, enhancing their natural tendency in mesenchymal differentiation and their capacity of differentiating into tenocyte lineage.

In conclusion, it is necessary to acquire a holistic view of stem cell regulation and reproduce the physiological conditions of stem niches that provide all the signals necessary for the maintenance of resident cells properties. For this reason, controlling oxygen tension from atmospheric (20%) to a more physiological level could be a good strategy to reach this goal. It is essential to note that tissue normoxia varies substantially in vivo, existing as gradients within tissue [258], whereby cell cultures could be influenced by oxygen concentrations [243] or time of exposure [259], and this means that the choice of adequate cell culture conditions is critical in order to develop effective cellular therapies.

### 2.3. Physical Stimuli

Tendon development, homeostasis, and regeneration following injury are based on the ability of tendon cells to biologically respond to externally applied forces. Tendons response to physiologic loading is strictly linked to its structure, cellular organization, and to the dynamic interactions between cells and their microenvironment [94]. 

**Figure 8:**
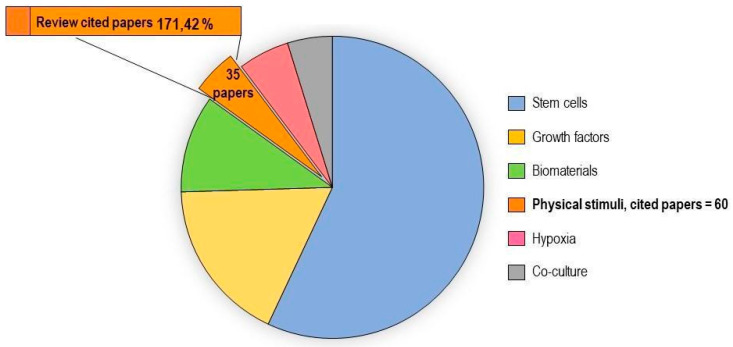
The orange section shows the percentage/number of papers available on the Scopus database obtained by combining key words in vitro stem cells tendon differentiation with physical stimuli. In the histogram, the relative percentage of the cited papers is expressed as well as the relative number in the legend.

Indeed, tendon cells are highly sensitive to mechanical inputs, and according to the magnitude, frequency, direction, and duration of the applied loads, they can adapt to their extracellular matrix in a catabolic or anabolic way [260,261,262]. Mechanical stimuli can also induce the activation of a biologic response that involves a complex set of pathways between the cell surface (ion channels, focal adhesion kinases, integrins, cytoskeleton) and the nucleus [94]. Just as physiologic loads are important to maintain tendon homeostasis [263,264], abnormal ones can cause injuries [265,266,267]. The study of tendon mechanobiology is essential to understand both the pathophysiology in tendon disease and the benefits of controlled applied loading during tendon healing and regeneration [94]. In fact, the bibliographic production on in vitro physical stimuli is still increasing (Figure 8).

Tendon tissue engineering strategies are largely scaffold-based, relying on decellularized structures, polymers, and/or gels that, mimicking the extracellular matrix environment, are able to provide an initial supportive structure to which mechanical loads can be applied. Choosing the scaffold with an appropriate mechanical behavior, e.g., stiffness and elasticity, a given load is delivered to the seeded cells. Scaffold mechanical properties have to match specific biochemical features such as bioresorbability and bioavailability to promote new tissue formation at the same time when implanted in vivo [166]. In this context, a bioreactor can act as a system that is able to recreate in vitro a suitable culture environment, which mimics the in vivo dynamics experienced by cells during tendon maturation, allowing cellular proliferation/differentiation and matrix production. Bioreactors for tendon tissue engineering require specific basic components such as an actuating system and a culture chamber, which provide, respectively, a construct’s mechanical stimulation and a controlled culture environment; continuous loading monitoring plus a feedback actuating system have been also described [268]. In this sense, both biopolymer scaffolds and bioreactors are complementary paradigms of tissue engineering, both converging to develop highly predictive in vitro biomimetic systems to study tendon regeneration and healing strategies.

Bioreactors can provide a given physical stimulus by direct or indirect modes. Indeed, tensile strain can be delivered directly by applying a cyclic and programmable load to the scaffold system, aiming to mimic in vitro the biomechanical environment of tendon tissue. Alternatively, a given strain indirectly provided to cells can be achieved by using different physical stimuli such as magnetic fields or acoustic waves. Based on these concepts, several custom-made bioreactors have been developed [202,263,264]. Two of these have become commercially available such as the LigaGen system (www.tissuegrowth.com/) or The Bose^®^ElectroForce^®^BioDynamic^®^ system (www.bose-electroforce.com). Commercial bioreactors are well designed even if they cannot meet all the specific requirements such as an easy and rapid scaffold fixation operation, adequate number of in vitro duplicates, or the necessity of a reduced amount of medium in the culture chamber [268].

Several in vitro studies are described using custom-made bioreactor systems including mechanical stimulation bioreactors. Compared to static, planar, and culture, scaffold-based approaches that undergo specific strain stimulation display more elongated cellular morphology and increased cell density. Moreover, compared to a load-free culture environment, a bioengineered scaffold can show up to a 9-fold increase in the cell number after 2 weeks of cyclic stretching [269]. On the contrary, it has been demonstrated that after 4 weeks in static conditions, tenocytes lose their typical elongated shape, becoming rounded, and the collagen fibers appear more crimped [270]. Tensile loads can deliver to the cells specific input to increase collagen synthesis with spatial organization along the stress direction and provide protection from collagenase [261,271,272]. Mechanical stimulation promoted the formation of bundles with parallel collagen fibrils, upregulating proteoglycans (decorin, biglycan, fibromodulin, and fibronectin) in the extracellular matrix [273,274]. The formation of collagen fibers along the direction of loading also results in an enhancement or optimization of the mechanical properties of the bioengineered tissue, such as stiffness, elastic modulus, maximum tensile stress, and maximum load [275,276,277].

The gene expression of tenogenic markers is also positively influenced by cyclic strain. For example, collagen type 1 expression under dynamic conditions has been reported to be three times higher than static conditions after 2 weeks of culture [278]. Furthermore, mechanical stimulus has been reported to orchestrate tenogenic differentiation upregulating Scleraxis, a helix–loop–helix transcription factor specific for tenocytes and their progenitors and particularly responsive to mechanical inputs [165,279,280,281]. Consistently, recent studies a showed reversible loss of Scleraxis expression after a gradual and temporary loss of tensile strain [282]. Another study demonstrated the upregulation of collagen type 1, collagen type 3, and Tenascin-C in human marrow stromal cells encapsulated in an oligo (poly (ethylene glycol) fumarate (OPF) hydrogel and cultured under cyclic tensile strain (10%, 1 Hz, 3 h of strain followed by 3 h without) for 21 days [283]. Additional data showed that only 24 h of moderate cyclic axial stretching (2% strain, 1 Hz) promoted the tenogenic differentiation and tendon matrix synthesis by equine adipose-derived mesenchymal stromal cells seeded on decellularized tendon matrix scaffolds, upregulating collagen type 3, decorin, Scleraxis and Tenascin-C [284]. Moreover, it has been reported that an intermittent cyclic tensile strain (10% applied strain, 1 Hz, 10 min every 6 h) enhanced the proliferation and tenogenic differentiation of human bone marrow-derived mesenchymal stem cells cultured in anisotropic collagen–glycosaminoglycan (CG) scaffolds, via time-dependent activation of ERK 1/2 and Smad 2/3 pathways. Cyclic strain promoted the activation of tendon-related (Tenascin-C, Mohawk and Scleraxis) and extracellular matrix biosynthesis-related genes (collagen type 3, Decorin, COMP) [285]. The effect of mechanical stimulation was further investigated on human embryonic stem cells-derived mesenchymal stem cells seeded on a collagen–silk scaffold. It was found that dynamic mechanical stimulation directed cells into a tenocyte-like morphology, expressing tendon-related markers (collagen type 1 and 3, Scleraxis) and other mechanosensory molecules (cilia, integrins, and myosin) [166]. However, the frequency of stimulation appears to be important in human tendon tissue engineering [276].

Moreover, also the percentage of strain applied can have different effects on tenogenic differentiation. For instance, tenocytes cultured for 12 days upon poly(glycerol-sebacate) (PGS) sheets under 6% cyclic strain exhibited a tendon-like gene expression profile compared to 3% and 0% strain groups [286], while uniaxial cyclic tensile stretching at 8% strain exclusively induced tenogenic differentiation of human bone marrow-derived mesenchymal stem cells, with protein and gene expression comparable to primary tenocytes [287]. On the other hand, constant strain has been found to negatively affect tendon diameter [288], also inhibiting cell proliferation and increasing apoptosis, potentially through the increase of heat shock protein (HSP)-72 expression [289]. In conclusion, maximum load, frequencies and cyclic strain are all parameters that have to be taken into account in order to achieve a highly predictive in vitro tendon-like bioengineered system. However, despite the importance of the choice of proper bioreactor device and operative parameters, this aspect has to be strictly merged with the scaffold mechanical properties. Indeed, it has to be underlined that any mechanical input can only be effectively delivered to a scaffold (i.e., to the cells on board) by taking into account the mechanical behavior such as the stiffness and elasticity of the specific scaffold chosen. In this sense, scaffold characteristics and mechanical performances have to be strictly selected and adapted to the bioreactor device.

Specific load to cells can be delivered using bioreactors designed with magnetic fields or shock waves. These bioreactors are designed for tendon tissue engineering because both electromagnetic field and shock waves are non-invasive therapies [290] and directly applicable in vivo to the injury site for the treatment of inflammation response, post-surgery re-tears [291,292] or tendinopathies [293,294,295,296]. Even if both therapies have been reported to have biological effects on impaired tendon tissue in vivo, including the local release of angiogenic factors and neovascularization, the differentiation of mesenchymal stem cells, and reduction in inflammatory mediators, the mechanism that induces regenerative and tissue-repairing effects in vivo is still under debate [297].

Magnetic bioreactor in vitro systems mainly provide a specific stress when the scaffold is designed with magnetic nanomaterials that are capable of responding to the applied magnetic field. In this sense, magnetic nanoparticles can act as magnetic actuators that are able to induce a specific strain that can trigger intracellular pathways or the release of biochemical signals [298]. Alternatively, magnetic particles can also be used as mechanotransduction platforms that are able to transmit forces to the cells in a localized manner, enabling the downstream activation of key tenogenic signaling pathways [299]. As mechanical bioreactors require scaffolds with proper elastic properties, Alternate Magnetic Force (AMF) bioreactors must be implemented with magnetic scaffolds and fabricated with biomaterials incorporating magnetic nanoparticles, resulting in magnetically responsive systems that are able to remotely boost cell mechanotransduction pathways [300,301]. The combination of magnetic fields and magnetic nanoparticles embedded within a 3D scaffold can create transient physical forces that are transferrable to cells present in the scaffold in close proximity to the nanoparticles, promoting the activation of signaling pathways involved in tendon development, homeostasis, and repair [302]. For instance, 3D printing technology has been used to fabricate an aligned fibrous structure of starch with poly(ε-caprolactone) (SPCL) carrying iron oxide magnetic nanoparticles and human Adipose Stem Cells (hASCs). The magnetic stimulation, obtained by an external magnetic field, promoted hASCs tenogenic differentiation, as confirmed by the expression of tendon markers and a collagenous tendon-like matrix [303]. More recently, magnetically responsive fibrous scaffolds have been obtained with an aligned electrospun thread of PCL and cellulose nanocrystals coated with iron oxide magnetic nanoparticles. Magnetomechanical stimulation of hASCs promoted their tenogenic commitment with higher degrees of cell cytoskeleton anisotropic organization, increased expression of tendon-related markers, and a pro-healing inflammatory gene profile, compared to unstimulated samples [304]. Moreover, magnetic nanoparticles can be attached to the cell membrane, and specific receptors can be activated using Alternating Magnetic Fields (AMFs) [298,305,306]. As a consequence of these simulations, a mechanotransduction event is obtained, evidencing the ability of cells to respond to mechanical stimuli using biochemical signals [307] Specific receptors on the cell membrane can be tagged with magnetic nanoparticles and then mechanoactivated with remote magnetic fields [308,309]. This approach was explored to induce the tenogenic differentiation of hASCs. The Activin receptor type IIA (ActRIIA) in hASCs was targeted with anti-ActRIIA functionalized magnetic nanoparticles and externally activated using an oscillating magnetic bioreactor. The results showed hASCs commitment into the tenogenic lineage via activation of the TGF-β/Smad2/3 signaling pathway [310].

Shock Waves bioreactors (SW) use transient short-term acoustic pulses with high peak pressure and a very short rise time to peak pressure (nanoseconds with short pulse duration) generated with a piezoelectric device. A combined treatment of shock waves and tenogenic medium (basal medium supplemented with 50 ng/mL of human insulin-like growth factor-1 and 10 ng/mL of human transforming growth factor β1) improved the differentiation of hASCs toward tenoblast-like cells, as evidenced by the upregulation of specific tendon markers, spindle-shaped cell morphology, and extracellular matrix fiber deposition [311]. Moreover, waves enhanced the functional activities of injured tendon-derived tenocytes, such as proliferation and migration [312], or they seemed to accelerate the induced differentiation of human tendon-derived stem/progenitor cells [313].

The above highlights the importance of a dynamic culture for tendon tissue engineering strategies where an appropriate pattern of mechanical stimulation plays an important role in the design of engineered tendon-like in vitro models. On the other hand, it is also evident that a dynamic cultivation can be obtained when taking into account scaffold composition and design, fabrication, and functionalization. Only through consideration of these aspects can the physical input can be effectively delivered to the cells and a biological response generated. In this sense, the mathematical modeling of the force distribution along the tridimensional scaffold after a specific load or force applied may further help the understanding of the biological output [202,314].

### 2.4. Biomaterials

**Figure 9:**
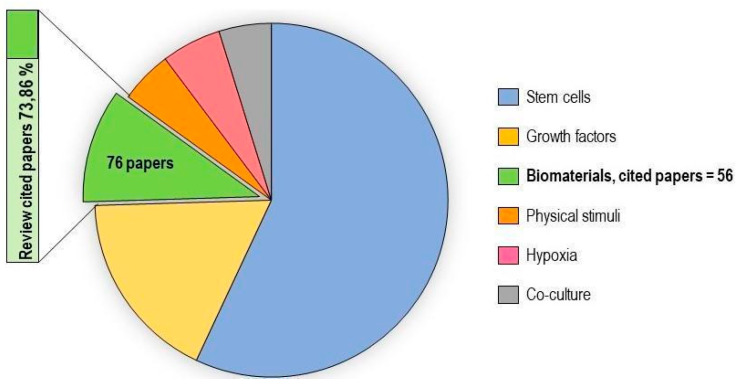
The green section shows the percentage/number of papers available on the Scopus database obtained by combining key words in vitro stem cells tendon differentiation with biomaterials. In the histogram, the relative percentage of cited papers is expressed as well as the relative number in the legend.

The design of a tissue regeneration matrix is based on two essential aspects: the material that constitutes it and the structure it should have. The ideal support matrix, “scaffold”, should possess optimum cell compatibility and should not draw out an inflammatory response or demonstrate immunogenicity or cytotoxicity [315,316]. Moreover, the scaffold must be bioresorbable, so that its by-products are eliminated through natural metabolic pathways in the human body with no residual side effects [315]. In particular, the scaffold designed for tendon tissue engineering must mimic the architecture of the native healthy tissue and compensate for its mechanical properties [315]. Three features of the tendon-like scaffold are crucial for tendon tissue engineering; the scaffold should be teno-inductive (capable of inducing the cell differentiation toward the tenogenic lineage), teno-conductive (support tendon growth and promote the ingrowth of surrounding tendon), and capable of teno-integration (integrate into surrounding tendon) [316]. The teno-inductive potential of the tendon-like matrix will be taken into consideration, since only in vitro studies will be discussed and described in Figure 9.

#### 2.4.1. Materials

Biomaterials play a pivotal role in scaffold fabrication providing three-dimensional templates and synthetic extracellular matrix environments for tissue regeneration. To fulfill the diverse needs in tissue engineering, various materials have been exploited. Polymers, materials widely used for scaffold design, are divided into two different categories: natural and synthetic. Polymers and their applications are crucial in tendon tissue engineering.

(1) Natural Polymers

Polymers of natural origin, such as collagen, silk, and chitosan, represent an interesting material choice for mimicry of the natural structure of a tendon and its properties. Collagen may be considered a good platform for tendon repair and reconstruction, since it represents the major component of the tendon and is characterized by its good biocompatibility properties [317]. For this reason, many researchers have focused on producing scaffolds with collagen alone or mixed with other molecules such as proteoglycans [318,319]. Despite the attraction offered by its biocompatibility, the main drawbacks of collagen scaffolds lie in their unsuitable mechanical properties linked to rapid degradation kinetics and poor structural stability as well as potentially their immunogenic character due to animal origin [320]. Alternative materials have been proposed for tendon reconstruction such as silk, which is a fibrous material secreted by spiders and by the caterpillars of certain butterflies (caterpillar of the mulberry bombyx) [321,322]. Silk possesses exceptional mechanical properties in terms of strength, toughness, and elasticity, making it popular in the field of tendon tissue engineering [323]. However, when used alone, silk does not allow sufficient cellular attachment or growth [322,323], forcing its use in combination with other materials that share similarity with native tendon ECM to improve its bioactivity [324,325]. Chitosan is another natural polymer that has been identified as a promising candidate for tissue engineering, since it shares many structural similarities with glycosaminoglycans (GAGs) present in native tendon ECM [326,327]. It is characterized by its biocompatibility, biodegradability, antibacterial capacity, and non-toxicity [328]. Despite these advantages, the high stiffness of chitosan membranes makes them challenging for applications in tendon tissue engineering due to their low mechanical properties [327].

(2) Synthetic Polymers

Another attractive material candidate for tendon tissue engineering is the synthetic polymers. This is due to their high flexibility and reproducible mechanical properties when compared to natural ones. The interest in bioresorbable synthetic polymers lies in the possibility of modulating their properties by varying their chemical composition and their structure, for example by choosing a particular molar mass or crystallinity or by combining two polymers with different characteristics. On the other hand, these polymers are mostly inexpensive, can be scaled industrially, and are thermoplastic, making them moldable and allowing the development of a wide variety of different structures [317,329,330]. Synthetic materials are common in tendon tissue engineering and belong mostly to aliphatic polyesters such as polyglycolic acids (PGA), polylactic acids (PLA), and polycaprolactones (PCL), as well as their copolymers poly (lactic-co-glycolic) acids (PLGA) and poly (lactic-co-caprolactone) acids (PLCL) [329,331,332,333,334]. Other materials are also used such as Poly (ester urethane) urea (PEUUR) [335,336], polyurethane (PU) [337], and polyethylene oxide (PEO) [338,339,340].

#### 2.4.2. Scaffold Fabrication

Different traditional techniques have been used to fabricate scaffolds for tendon regeneration including sponges [341,342,343], freeze-drying [323,324,344,345], supercritical fluid processing [346,347], extruding [348], electrochemically aligned collagen [319,349,350], and electrospinning [322,325,332,333,351,352]. Recently, 3D bioprinting has emerged as an novel technique in the field of tissue engineering aiming at fabricating organized scaffolds with complex shapes [352,353,354,355,356]. In comparison with the conventional techniques above listed, 3D scaffolds possess a better controllable pore size and geometry as well as with good mechanical properties that are easily designed and fabricated using 3D printing to mimic the ECM of the tissue to be regenerated [357]. Many 3D printers are now available to fulfill the requirements of the ECM to be fabricated with heterogeneous structures or interfaces [358,359].

To date, amongst these techniques, considering the morphology of native tendons and their oriented fibrillary structure, the electrospinning process is selected for its capability to obtain fibrous constructs, with an average diameter ranging between the nano- and the micro-scale, aimed at resembling the architecture of the native tendon ECM [360]. The scaffolds produced via electrospinning are considered and discussed. Electrospinning is a shaping technique that has become very popular in the fields of biomaterials and tissue engineering due to its potential to produce fibers of micrometric or even nanometric diameters via the application of an electrostatic field [361,362,363,364]. In detail, when a high voltage is applied on the polymer solution, a pendant drop will be formed at the needle tip. Two electrostatic forces will be applied on the formed pendant drop and are divided into electrostatic repulsion and Columbic forces that are formed between the surface charges and exerted by the external electric field, respectively [361,362,365]. The obtained structures have the advantage of being three-dimensional and are increasingly comparable to real tendon structures. The microstructure of the produced support matrices possesses a large specific surface area that aims to increase the cell adhesion capacity, promote protein adsorption, and present more anchoring sites for receptors on the cell membrane [363,365,366]. Such microporous architectures are in fact close to the morphology of the collagen fibers constituting the tendon ECM, allowing a biomimetic character to strongly promote cell colonization and differentiation [190,318,325,367]. The produced fibers can be adjusted and modified by varying the polymer (concentration, conductivity, solvent) and electrospinning process parameters (flow rate, voltage, collector, distance between needle and collector) [190,318,328,368,369]. Different synthetic polymer scaffolds can be fabricated by optimizing electrospinning process parameters for tendon tissue engineering. It is of great interest to understand how scaffold parameters including fiber alignment and diameter size affect cell behavior by controlling and modulating their proliferation and differentiation. The fabricated synthetic scaffolds can have a fiber diameter ranging from nanometers (<1 μm) to micrometers (>1 μm) with fiber alignment ranging from randomly oriented fibers to aligned fibers arranged in parallel. Fiber alignment is considered as a key factor for mimicking tendon ECM and can be modulated and optimized on the basis of the collector used. Randomly oriented fibers can be obtained using a ground collector while those with aligned topography can be fabricated using a rotator drum/mandrel [190,338,340,350,370] and parallel copper electrodes [301]. By using an electrospinning technique, different scaffold shapes for tendon tissue engineering have been fabricated such as aligned meshes [190,328,335,339,340,350,370], bundles [371], multilayer scaffolds [368], and stacked and braided scaffolds [166,367,372]. In the biomimetic concept of tendon-like ECM, the effect of fiber orientation and diameter size are evident on the mechanical properties of the produced electrospun scaffolds (Table 1) as well as on the cellular biological response (Table 2).

#### 2.4.3. Tendon Biomimetic Scaffold Structure and Mechanical Properties

Taking into consideration the native tendon structure characterized by aligned collagen fibers, electrospun scaffolds with aligned fibers seem to be an interesting target for mimicking tendon ECM (Table 1). Full et al. produced PLGA/ColI/PU random and aligned fiber scaffolds using a static and rotator mandrel collector at 300 rpm, respectively. Two different PLGA compositions were used: 85:15 and 50:50 for lactic acid/glycolic acid, respectively. The mechanical properties in terms of Young’s modulus and tensile stress were higher in the aligned fibers with respect to random fibers and especially for those fabricated with PLGA (50:50) ColI/PU [350]. Moffat et al. produced PLGA scaffolds with aligned fibers using a rotating ground collector at a speed of 20 m/s. The elastic modulus and the ultimate stress as well as the strain were three and 10 times higher than that of random fibers [371]. Moreover, Russo et al. produced electrospun PLGA fleeces with randomly oriented and highly aligned fibers using a cylindrical rotator drum at a rotational speed of 100 and 1000 rpm, respectively [190], while Zhang et al. [340] produced CS/polylacticacids (PLLA)/Gelatin/PEO nanofibers with randomly oriented and aligned fibers using a ground collector and rotating drum at 1000 rpm [340], respectively. The produced PLGA and CS/PLLA/Gelatin/PEO fibers with aligned fibers again showed better mechanical properties compared to the random ones [190,340]. Zhang et al. [338] produced PLLA/PEO mats loaded with trichostatin A (TSA) with randomly oriented and aligned fibers using a grounded and rotator drum with 1000 rpm speed, respectively. The ultrastructure analysis of the produced mats showed that the insertion of TSA into the PLLA/PEO led to thinner fiber. The mechanical properties of the aligned fiber groups appeared to be superior compared to the random ones, since the tensile strength and Young’s modulus of the aligned fibers were about 5 and 10 times higher than those of the random ones [338]. Moreover, Nitti et al. produced CS-PEO random and aligned nanofibers using static and rotating drum collectors, respectively. The rotation of the collector varied between 800 and 2500 rpm [339]. The produced aligned nanofibers showed an improvement in the mechanical properties in terms of Young’s modulus, stress at break, and elongation at break by increasing the rotational speed of the collector that allows obtaining highly aligned fibers with respect to random nanofibers [339]. Yin et al. [370] produced PLLA aligned fibers using a rotator mandrel at a rotational speed of 4000 rpm, and they demonstrated that the mechanical properties of the aligned in terms of failure force, Young’s modulus, and stiffness were enhanced by 11, 36, and 50 times, respectively, compared to random fibers [370]. Lee et al. [337] produced PU nanofibers with a diameter of about 657 nm with aligned and randomly oriented fibers. Confirming the previous results, they also demonstrated that fiber alignment improved the mechanical properties of the scaffolds in which Young’s modulus and the ultimate strength were 5 and 3 times higher compared to the randomly oriented fibers [337]. Some researchers have considered that the electrospun sheet matrix should not be considered as a 3D environment, so it is necessary to modify the electrospinning set-up devices in order to obtain an improved scaffold with 3D structure. For this reason, Orr et al. [366] fabricated aligned and random PCL multilayer scaffolds by combining ceramic magnet and parallel copper electrode methods using a reservoir containing distilled water. The assessment of mechanical properties revealed that fiber alignment of the PCL multilayer scaffold enhanced the mechanical properties (elastic modulus, yield strength, and yield strain) compared to the random PCL multilayer scaffolds [366]. Sensini et al. [371] produced two different electrospun bundles with aligned fibers using two different composition ratios 75:25 and 50:50 for PLLA/Col I. The single fiber and the bundle diameter size were unaffected while better mechanical properties were obtained with bundle PLLA/Col I (75:25) [371]. Moreover, Rothrauff et al. [367] designed braided and stacked scaffolds made up of PCL and PLLA. Although the fiber diameter size of the produced electrospun aligned polymer sheets was similar, higher Young’s modulus and stiffness values were obtained with the stacked scaffolds, especially the PLLA ones [367]. Wu et al. [373] fabricated 3D electrospun yarns made up of PCL mesh with aligned fibers with a final diameter of 209 μm. When compared to the 2D aligned and random PCL mesh, 3D yarns exhibited higher ultimate tensile strength and Young’s modulus [373]. Moreover, Tomàs et al. [304] proposed the fabrication of PCL yarns with dodecanethiol nanoparticles (DT-NP) at different concentrations (0%, 2.5%, and 5%). The resulting PCL/DT-NP meshes, after being collected onto the surface of a grounded liquid bath, were pulled by a roller at a constant speed to form the threads. The resulting threads showed an increase in their diameter by increasing DT-NP concentrations (44.9 μm vs. 184.9 μm for PCL and PCL/DT-NP5). Twelve threads were twisted to form the different yarns in which their diameter size was ranged from 313 to 346 μm. It was noticed that the incorporation of DT-NPs within the PCL constructs had a positive impact on their mechanical properties since increasing DT-NPs content (from 0% to 5%) increased in turn the Young’s modulus (from 12 to 22 MPa), strain at break (from 3.4 to 4.2 mm·mm^−1^), and the stress (from 2.9 to 4.8 MPa) [373]. Czaplewski et al. [374] fabricated braided submicron aligned fibrous scaffolds (BSMF) with a diameter of about 970 nm made up of a PCL and PLLA blend with different PCL/PLLA ratios (100:0; 75:25; 50:50, and 25:75) and a varying number of stitches per inch (8, 12, 16, 20, and 24). The mechanical characterization revealed that increasing PLLA content influences negatively on the mechanical properties in terms of Young modulus, ultimate strength, and yield strength. Moreover, it was noticed that braided scaffolds with 8 SPI (characterized by a braiding angle of about 67°) increase the Young’s modulus of the scaffolds compared to other studied stitches per inch (SPI) values [374]. As mentioned before, the electrospinning technique allows producing a fibrous matrix with a fiber diameter size in the nano- and micro-range. Few studies have evaluated the effect of changing fiber microarchitecture on scaffold characterization, which could be of a great concern in tendon tissue engineering. Erisken et al. [375] produced PLGA fibers with diameter of 320 nm, 680 nm, and 1.80 μm by using different polymer concentrations to mimic tendon ECM during the different stages of the healing process. In terms of mechanical characterization, they found that while the tensile modulus increased by increasing the fiber diameter size, ductility and elongation at break decreased [375]. In another study, Kim et al. [376] produced four PCL mats with different diameter size in the range of 0.11–3.43 μm. They found after mechanical characterization that Young’s modulus, ultimate tensile strength, and strain at break increased consequently by 4, 7, and 9 times when increasing the fiber diameter size [376]. In another study, Cardwell et al. [336] fabricated different poly (ester urethane) urea scaffolds with either random and aligned fibers possessing different diameter size <1 μm, >1–2 μm<, and >2 μm. They found that smaller fiber diameter side exhibited a higher degree of alignment compared to the larger ones [336]. Thus, the electrospun aligned fibers in the micrometer range, due to their distribution along the longitudinal axis of the uniaxial tendon, possess better mechanical properties compared to randomly oriented fibers and those in the nanometer range, making the scaffold stiffer in the direction of applied force, allowing them to support the damaged tissues during tendon regeneration.

#### 2.4.4. Teno-Inductive Potential of Electrospun Produced Materials

Tendon biomimetic scaffold efficacy is proven by testing its teno-inductive potential on the seeded cells, which can be modulated by the underlying substrate topography and fiber diameter. These parameters may influence cell adhesion, proliferation, spreading, organization, and differentiation toward the tenogenic lineage, and they may also control the deposition of ECM.

Different cell sources have been used to assess the teno-inductive potential of scaffold topography (Table 2). Studies have assessed the effect of fiber alignment of electrospun scaffolds by producing fibers with aligned and random orientation on cell teno-differentiation. In a study conducted by Moffat et al. [370], they cultivated human rotator fibroblast-like cells onto PLGA scaffolds with random and aligned fibers under static conditions. After 1-day culture, the cells started to elongate along the fibrous matrix of aligned fibers and maintained the cell shape and organization up to 14 days of culture. After this culture period, no differences were noted in cell proliferation and adhesion between aligned and random fibers. Interestingly, the bio-hybrid PLGA aligned fiber–cell maintained their mechanical properties in vitro compared to the random ones and allowed teno-differentiation characterized by the upregulation of tendon-related markers and the deposition of Col I and Col III as ECM [370]. In a similar study, Zhang et al. [340] compared the effect of fiber alignment of CS/PLLA/Gelatin/PEO on human iPSC-MSCs without applying any mechanical stimuli [340]. Similar results in terms of cell proliferation, adhesion, and morphology as well as the upregulation of tendon-related gene markers as Moffat et al. [370] on the aligned fibers were obtained in this study compared to the random ones, confirming the importance of fiber alignment in cell guidance and teno-differentiation [340]. Leung et al. [328] cultivated human primary BMSCs onto aligned and random fibers of CS-PCL scaffolds under static conditions. Even if no differences in cell adhesion, fibroblast cell morphology, and Tnmd gene expression were detected between random and aligned scaffolds, Col I and Col III were upregulated only on the aligned fibers [328]. Moreover, Yin et al. [369] fabricated aligned and randomly oriented PLLA scaffolds on which they evaluated human TPSCs potential [369] under static conditions. After 3 days of culture, they showed that the transcription factor genes Scleraxis and eya2, and the matrix gene Col XIV, were expressed significantly in aligned fibers, while Ocn and Alp expressions were significantly upregulated in the randomly oriented ones, implying that aligned fibers promote cell differentiation toward the tenogenic lineage while those randomly oriented stimulate it to the osteo-lineage [369]. Russo et al. [190] assessed the effect of electrospun PLGA fiber alignment on AECs. In contrast to the previous results obtained in terms of cell proliferation and adhesion, the fiber alignment seems to affect cell activity, since the DNA quantity and cell proliferation rate decreased onto the aligned fibers compared to the random ones. However, interestingly, after only 48 h of culture, the aligned fibers were able to induce an early epithelial–mesenchymal transition (EMT) on AECs, revealing the downregulation of epithelial markers (Cytokeratin-8) and upregulation of the mesenchymal ones (Snail, Vimentin, and α-SMA). This AEC’s EMT was followed by their teno-differentiation, which was detected after 48 h up to 28 days without using any tenogenic differentiation media. These cells expressed mature tendon related genes (Tnmd and Col I) and Col I protein only on the aligned fibers, which became, after 28 days of culture, expressed extracellularly, forming a sort of ECM [190]. Additionally, to the effect of fiber topography on AEC tenogenic differentiation, long term co-culture has been conducted up to 28 days by using fetal tendon explants [184] with bio-hybrid AEC-PLGA fleece. The results showed that AEC tenogenic differentiation accelerated to take half the time compared to PLGA fleeces cultured only with AECs. AECs represent an important cell source to evaluate the teno-inductive potential of electrospun aligned fibers since their epithelial origin allows the verification of the mechanisms supporting epithelial cells (cuboidal in its morphology and negative to Col I expression) differentiation toward mesenchymal cells of the tenogenic lineage (spindle-like morphology) [190]. Based on previous observation of reduced expression of histone deacetylases (HDACs) in tendon stem/progenitor cells (TSPCs) cultured on aligned fibers [367], Zhang et al. [338] proposed a strategy to enhance the tenogenesis effect of aligned fibers by developing epigenetic bioactive PLGA scaffolds with well-aligned fibers incorporated with Trichostatin A (TSA), an HDAC inhibitor molecule. From the obtained results conducted under normal static culture, Zhang et al. concluded that even if no difference has been detected in cell proliferation, cells seeded onto aligned nanofibers incorporated with TSA showed higher elongated morphology with a higher expression of ScxGFP protein (70% vs. 35% aligned nanofibers). Additionally, aligned nanofibers with TSA enhanced the expression of tenogenic proteins (Col I, Col V, Tnmd, and Epha4), upregulated the expressions of tendon-related gene markers (Scx, Mkx, Eya1, Eya2, Six2, HoxA11 and Egr1) and of HADC 3 and 4, while they downregulated HDAC 1 compared to other groups [338]. Moreover, Lee et al. [337] investigated the effect of human ligament fibroblast on aligned and randomly distributed PU fibers under 5% strain for 24 h at a frequency of 12 cycles/min. They observed that while no histological differences were observed between both culture conditions (static and dynamic), an increase in collagen ECM deposition was revealed in the aligned fibers subjected to cyclic culture conditions [337]. Moreover, Subramony et al. [377] evaluated the combined effect of dynamic culture condition (1% strain at 1 Hz for 90 min for 2 days) and PLGA fiber alignment on human MSCs differentiation. They found that while cells acquired an elongated morphology on aligned fibers under both culture conditions, cells seeded onto randomly oriented fibers became elongated under only dynamic culture conditions. Col I and Col III were upregulated in the aligned scaffolds subjected only to mechanical stimuli. An upregulation in Col III, fibronectin, tenascin-C, and a downregulation in Scx has been shown in cells seeded onto the aligned fibers under dynamic culture conditions [377]. Wu et al. [373] evaluated the effect of woven aligned fabric scaffolds on human tenocytes and human ADSCs alignment, proliferation, and enhancement of their tenogenic phenotype compared to the random and aligned meshes [373]. When cultured with human tenocytes (HT), all scaffold types expressed Tnmd and Col I proteins with a significant increase in tendon-related gene markers in woven fabrics. When seeded with human ADSCs, there was a significant increase in cell proliferation, infiltration, and tendon-related gene expression of Scx, Col I, and Tnmd in the woven fabrics only [373]. Moreover, they performed co- and tri-culture of HADMSC with HT or human umbilical vein endothelial cells (HUVEC) on woven fabrics, and they found that all tendon-related markers (Scx, Tnmd, Tnc, Col I, and Col III) were upregulated together with the vascular endothelial growth factor A (A-VEGFA) and angiopoietin 2 (ANGPT2) in the tri-culture system compared to other groups. They also conditioned the tri-cultured constructs with dynamic culture and demonstrated that dynamic stretch promoted collagen secretion and tenogenic differentiation compared to the static condition [373].

Other authors have produced 3D scaffolds made up of multilayered aligned electrospun fibers from which they fabricated braided and stacked scaffolds. Rothrauff et al. [367] evaluated the effect of multilayered PCL–PLLA scaffolds of aligned electrospun nanofibers of two designs, stacked or braided scaffold with human BMSCs under static conditions [367]. In this study, it was demonstrated that both 3D scaffolds supported the expression of tenogenic markers with a greater effect noticed on braided ones. Conversely, higher cell infiltration and distribution was observed in stacked scaffolds, resulting in enhanced cell proliferation, collagen, and sulfated glycosaminoglycan contents [367]. In another study, Orr et al. [368] evaluated the effect of multilayer PCL scaffolds of aligned and random electrospun microfibers on human ADSCs, without any mechanical stimulation, where they did not find any difference in cell proliferation, glycosaminoglycan, Col I, and Col III content between the aligned and random groups. Moreover, both random and aligned fibers were able to express the tendon-related genes with higher expression levels in the aligned fibers. Mechanically, the cellular biohybrid aligned scaffold showed a significant increase in mechanical properties, since the Young’s modulus and yield strength values after 28 days of culture were two times higher than those of the acellular scaffolds [368]. Other studies were conducted to evaluate the effect of 3D yarn scaffolds on cell activation. Czaplewski et al. [374] investigated the effect of fiber chemistry and braiding angle of aligned braided submicron fibrous matrix (BSMF) on the tenogenic differentiation of hiPSC-MSCs subjected to cyclic tensile stimulation (3 days under static condition then under 3% strain 25 Hz for 2 h/day for 7 days). Results showed that cells became fusiform in the PLLA scaffolds made up of 8 stitches per inch (SPI) compared to the 24 SPI. Moreover, while there was an upregulation of tendon- and osteogenic-related genes markers (Col I, Col III, Runx2, and Ocn) on PLLA scaffolds under static culture condition, cells cultured onto PLLA scaffolds under dynamic culture conditions downregulated the expression of osteogenic markers (Runx2 and Ocn). The lowest braided angle (67° corresponds to 8 SPI) favored cell elongation compared to 24 SPI (where cells were cuboidal) and downregulated the expression of Col I, Col III, Runx2, and Ocn after 10 days of culture under cyclic condition. It seemed that scaffolds braided with large angles (8 SPI) better supported iPSC-MSC tenogenic differentiation by upregulating the tendon-related genes and downregulating those of osteogenic lineage under cyclic culture condition compared to braided scaffolds with small angles [374]. In another study, Tomàs et al. [304] produced PCL yarns with 5% iron oxide nanoparticle (DT-NP) concentrations and studied their effect on human ADSCs under static and magnetic stimuli conditions [304]. These PCL yarns were able to upregulate tendon-related genes (Tnmd, Dcn, Col I a1, Col III a1, Tnc, Scx) with a significant increase in the expression of Scx and Tnmd under magnetic stimulation conditions. In addition, cells exposed to magnetic stimulation downregulated osteogenic-related genes (Runx2) after 11 days of culture. The magnetostimulation of hADSCs in the PCL/DT-NP5 tended to increase gene expression that induced tenogenic commitment on hADSCs while it decreased the expression of genes related with other lineages. They also demonstrated that the possible molecular mechanisms underlying the regulation of fiber alignment on the stem cell differentiation of hADSCs toward the tenogenic lineage may be mediated by the Yes-associated protein/Trascriptional Coactivator with PDZ-binding motif (YAP/TAZ) signaling pathways through which aligned fiber topography induced cell polarization and cytoskeleton tension to trigger this effect [304]. Other groups have studied the effect of fiber diameter on cell biological activities. In a study conducted by Erisken et al. [375], human rotator cuff fibroblasts were cultured under static conditions onto PLGA scaffolds with different fiber size [375]. The cells exhibited more aligned and elongated morphology with high levels of tendon-related gene markers (Col III, Col III, Col V, and Tnmd) on the larger fiber scaffolds while a high cell proliferation rate and production of tendon-like matrix (Col I and GAGs) were detected on the smaller fiber scaffolds after 28 days of culture [375]. In a similar study, Cardwell et al. [336] demonstrated that fiber diameter, not alignment of PEUUR scaffolds, affected the differentiation of C3H10T1/2 toward the tenogenic lineage [336]. They observed that cell density and proliferation decreased by increasing fiber diameter. Moreover, after 14 days of culture, cells underwent tendon/ligament differentiation by upregulating Col I and Scx markers on larger fibers, regardless of fiber alignment [336]. Similar results in terms of cell density and proliferation were also obtained by a study conducted by Bashur et al. [335]. However, in this study, it was noted that the expression of these markers (Tnmd, Col I, and Dcn) decreased with decreasing fiber diameter while Scx gene expression increased by increasing fiber diameter after 7 days of culture [335]. Lee et al. [378] tried to model the various stages of repair post tendon injury by preparing PLGA polymer meshes with randomly oriented fibers possessing different diameter (nano-1 (390 nm) < nano-2 (740 nm) < micro (1420 nm)), and they produced only aligned fibers with the nano-2 group to mimic the biological healing phase rather than the scar formation. It was observed that cell growth and collagen synthesis were enhanced on the randomly oriented nanoscale fibers compared to the micro-sized fibers. The organization of paxillin and actin on randomly oriented fibers was enhanced on micro-sized compared to nano-sized fibers, while the expression and activity of RhoA and Rac1, proteins from the Rho family of GTPases that were characteristic of the initial proliferative phase of wound repair were greater on nanofibers. In contrast, early cell organization was promoted by cell alignment accompanied by reduced cell growth and collagen production [378]. It can be noticed that microfibers, which resemble the tissue remodeling stage and where collagen fibers display a diameter and organization closer to those of healthy tendons, are preferable to maintain the tenogenic phenotype of tenocytes rather than nanofibers that seemed to recapitulate the early stages of tissue repair. Additionally, in vivo studies have been conducted with aligned and randomly oriented electrospun scaffolds from which it has been confirmed the effect of using aligned fibers on enhancing the tendon regeneration in rat Achilles tendon [338,340] and mouse skin models [369] (Table 3). Overall, the results showed that fiber alignment and diameter topography affect cell behavior and could be controlled and optimized to design a potential scaffold for tendon tissue engineering. In particular, the discussed results underscore the complex relationship between the subcellular geometry and global cell function. The activation of downstream signaling pathways depends on the cytoskeletal actin stress fibers and focal adhesion of the cells in a specific 3D environment. This, in turn, affects cell functions including adhesion, migration, morphology, proliferation, gene expression, and differentiation. In addition, co-culturing the bio-hybrid scaffold with other cell types or cultivating it under cyclic conditions allows the acceleration of further cell tenogenic differentiation and organization. However, the differences in the results clearly show the effect of fiber alignment and diameter on regulating cell teno-differentiation ability, which could be attributed to the differences in the physicochemical properties of the used polymers as well as to the used cell sources that greatly affect cellular response in vitro. Taken together, the electrospinning technique may represent a favorable technique to produce fibrous matrices with appropriate structural and mechanical properties, making them useful for tendon tissue engineering applications. It was confirmed that fiber alignment, diameter, and 3D structure of scaffold (yarn, knitted, stacked, braided) enhance cell activity by promoting their differentiation toward the tenogenic lineage while hindering other different lineages (i.e., osteogenic, cartilage).

### 2.5. Growth Factors

#### 2.5.1. A Lesson from the Role of Growth Factors In Vitro

Growth Factors (GFs) that are involved in tenogenesis and able to control progenitor cell biology belong to a number of different families including transforming growth factors beta (TGF-β1, TGF-β2 and TGF-β3), bone morphogenetic proteins (BMPs: BMP-12, BMP-13 and BMP-14), Fibroblast Growth Factor (FGF-2), vascular endothelial growth factor (VEGF), connective tissue growth factor (CTGF), platelet-derived growth factor (PDGF), and insulin-like growth factor 1 (IGF-1) [379,380,381,382]. The majority of data available to date on the teno-inductive roles of the different categories of GFs are derived from evidence collected during developmental and regenerative tenogenesis.

**Figure 10:**
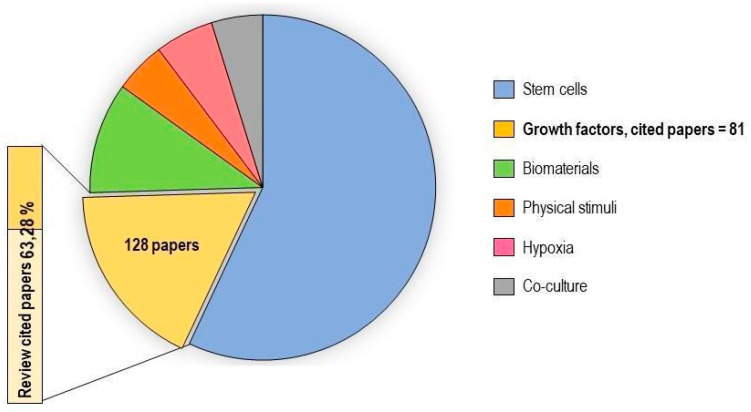
The yellow section shows the percentage/number of papers available on the Scopus database obtained by combining key words in vitro stem cells tendon differentiation with growth factors. In the histogram, the relative percentage of cited papers is expressed as well as the relative number in the legend.

GFs with roles in driving regenerative and reparative tenogenesis are synthesized and secreted by a wide variety of cells. These include inflammatory cells, platelets, fibroblasts, epithelial cells, vascular endothelial cells, and tendon progenitor cells. The GFs released in response to tissue damage bind to external receptors on the cell membrane, leading to intracellular pathways involved in DNA synthesis and transcriptional expression directly affecting multiple cellular processes including proliferation, chemotaxis, matrix synthesis, and cell differentiation, all being able to influence the healing cascade. In postnatal life, GFs begin to be activated from their storage inactive form, being orchestrated by a dialogue occurring across different cell compartments that contribute to tissue homeostasis [3,51]. In repair, tissue release of GFs is triggered, firstly, from the activated platelets straight after injury. This is followed by GF-driven initiation of the inflammatory cascade, recruiting inflammatory cells to the site of injury that, in turn, secrete additional GFs and amplify the inflammatory cascade. The stem/progenitor tendon cells situated next to the injury area are activated and themselves produce GFs. Further, mechanical loading placed on the injured tendon can further modulate GFs production and their paracrine release [1,3,51,383]. This evidence seems to suggest that a physiological tendon-inductive microenvironment requires multiple GFs over a specific temporal pattern and an optimized relative ratio [3,379]. This complexity is likely the explanation for the lack to date of reproducible GFs formulations for the induction of in vitro tenogenesis despite the consensus positions available for osteogenesis and chondrogenesis [51,384]. However, several efforts have been made to validate the in vitro delivery of GFs for tenogenesis, as demonstrated by the scientometric analysis (Figure 10). Their efficacy will be evaluated below taking into account the differences in GF category, stem/progenitor cells plasticity, stepwise tenogenic outcomes, and readout indices.

#### 2.5.2. TGF β

The transforming growth factor beta (TGF-β) pathway is the most recognized signaling pathway for tendon development [380]. It is active in all stages of tendon healing, and its expression is upregulated in differentiated tendon cells [115,382,384,385]. TGF-β induces extrinsic cell migration, regulates proteinases and cell proliferation, and stimulates collagen production. Moreover, its expression pathway in the human tendon is crucial in the tendon’s adaptation to mechanical loading [386]. In mammals, three different TGF-β isoforms, (TGF-β1, -2, and-3) of the 25 kDa homodimer are expressed, and in mice, the Tgfβ2^−/−^, Tgfβ3^−/−^ double knockout genes give rise to a distinct phenotype resulting in the loss of almost all tendons in the limbs, trunk, tail, and head [118]. TGF-β1 is a potent inducer of the tendon transcription factor Scleraxis (Scx) [114] and Mohawk (Mkx) expression [118]. In particular, during the development of tendons, it has been shown that the TGFβ-Scx pathway plays a critical role in the initial differentiation of tendons, followed by the TGFβ-Mkx pathway playing an essential role at the tendon maturation stage [120]. In vitro experiments demonstrating the TGF-β1 role on in enhancing tenogenic marker expression have been performed using tendon stem/progenitor cells (TSPC), mesenchymal stem cells (MSC), bone marrow-derived mesenchymal stem cells (BMSC), as well as in embryonic stem cells (ESC), [114,387,388]. Zhang B et al. [342] showed that TGF-β1 treatment induced the differentiation of rabbit BMSC to tenocytes seeded in a three-dimensional (3D) culture system based on a silicone chamber and collagen sponge scaffold. TGF-β1 supplementation on BMSC seeded on collagen sponge constructs for 3 days increased the cell viability and mRNA and protein expression levels of tendon-related marker genes, including Col I, Col III, Tnc, Scx, and Tnmd. Meanwhile, TGF-β1 inhibited the mRNA expression of PPARγ adipogenic marker and Runx2 osteoblast-specific marker differentiation. Furthermore, by using the BMSC–collagen sponge construct treated with TGF-β1 in a rat Achilles tendon in situ repair experiment, it was shown that enhanced structural and functional tendon regeneration was achieved [342].

In human adipose-derived stem cells (ASCs) culture, TGF-β1 supplementation in the presence of ascorbic acid (AA) to the culture medium was able to increase Scx genetic expression after 7 days of culture following by an increase of a collagen type I expression [389]. These results were in agreement with those obtained by Cheng et al. in human anterior cruciate ligament-derived stem cells (LSC) cultured with TGF β1 where collagen type I and type III, tenascin-c, fibronectin, and a-smooth muscle actin were significantly upregulated after treatment. However, simultaneous TGF-β1 with FGF-2 treatment increased the production of collagenous and non-collagenous extracellular matrix proteins, supporting the idea that this cell type is able to generate a different response depending on the cytokine input [390]. In canine adipose-derived MSC cells (ADSCs), TGF-β1 administration induced cell differentiation toward tenogenic lineage when in the presence of IGF1 in a high-density culture and in 3D co-culture with primary tenocytes. Cells treated with TGF-β1 and IGF1 expressed high levels of Scx, and Col I, Col III, Dnc, and Tnmd expression proteins after 7 days of culture. After 14 days, cells showed an expression of tendon-related markers to levels comparable with tenocytes. Treatment enhanced extensive formations of intercellular contacts and produced a well-organized extracellular matrix through the activation of the MAPK kinase pathway signaling members, adaptor proteins, Shc, and ERK1/2 [391]. Recently, it has been demonstrated that TGF-β, physiologically secreted from amniotic epithelial cells (AEC) and spontaneously accumulated during cell expansion, by inducing the epithelial–mesenchymal transition (EMT) process, allows human and ovine AEC cells to loss their epithelial morphology and to acquire a mesenchymal phenotype [191]. Treatment with a TGF-β signaling inhibitor (SB-505124) induces the complete reversion of in ovine AEC that experienced EMT, thus confirming the involvement of TGF-β1/Smad pathway in the process [191]. The production of TGF-β1 and its induction of the EMT process could represent the first event in the promotion of AECs differentiation toward the tenogenic lineage in a stepwise in vitro manner. It has also been demonstrated that oAECs have an in vitro ability to differentiate into tenocytes in allo and xeno co-culture with tenocyte explants and when exposed to teno-inductive scaffold topology [184,188,190]. A stepwise protocol using TGF-β1 to initiate tenogenic differentiation, followed by a combination of TGF-β/connective tissue growth factor (CTGF) to further maintain the teno-lineage, was also recently reported to better promote tenogenic differentiation in BMSCs [226]. It was found that in BMSCs, treatment with TGF-β1 alone firstly induced an in vitro elongated shape morphology and significantly increased Scx, Col I, Tnc, and Thbs4 within 3 days of treatment. However, only when in the presence of CTGF and TGF-β1 was elevated Tnmd mRNA and protein expression maintained at day 7 of BMSCs culture. The efficacy of this stepwise inductive in vitro approach by using TGF-β1 and TGF-β1/CTGF sequentially was further evaluated in a patellar tendon injury model establishing its effectiveness for the induction of tenogenic differentiation. Yin et al. [226] demonstrated that the TGF-β1 signaling pathway, through Smad2/3 activation, is essential for tendon differentiation in BMSCs and that it was dominant over chondrogenesis. Indeed, even if the expression of Sox9 increased at day 3, its expression decreased gradually during the optimized stepwise tenogenic induction protocol, and the mature chondrocyte marker Col II was undetectable in inducted BMSCs. TGF-β1 supplementations were also used to maintain the tenogenic differentiative status of equine tenocyte isolated from superficial digital flexor tendon (SDFT) and cultured in vitro by employing the 3D hanging drop technique and cultured as scaffold-free microtissue spheroids (3D) in low serum medium containing ascorbic acid and insulin. This was made evident by significant increases in the expression levels of pro-tenogenic markers Col II a1, Col III a1, Scx and Tnmd, as well as by enhanced levels of collagen type I and tenomodulin proteins. After TGF-β1 treatments, equine tenocytes showed a typical spindle-like morphology and when embedded in collagen gels, they became highly aligned with respect to the orientation of the collagen structure following their migration out from the microtissue spheroids [392].

Despite the volume of information on the use of TGF-β1 isoforms in inducing in vitro tenogenesis, few data are available on the other two isoforms, TGF-β2 and TGF-β3. Of note, Liu et al. [393] have recently demonstrated that treatment with endogenous microRNAs miR-378a, with its TGF-β2 binding target, impaired tendon healing by suppressing collagen and extracellular matrix production in miR-378a knock-in transgenic mice. They also showed that in vitro tenogenic differentiation was suppressed from miR-378a in TDSCs isolated by transgenic mice [393]. These results seem to support the TGF-β2 role in tendon differentiation and development and are in agreement with other reports in which it was demonstrated that in vitro treatment with this TGF-β2 isoform induced tenogenic Col I and Scx expression in mouse C3H10T1/2 stem cells [114]. Consistently, Havis et al. [115] showed that the same typology of cell exposed to TGFβ-2 or TGFβ-3 upregulated Scx and Col I a1 gene expression, and in contrast, TGF-β ligands significantly decreased the relative levels of the cartilage marker Sox9. Moreover, by investigating the molecular mechanism involved, this study demonstrated that the TGF-β2 ligand has the ability to direct mouse mesenchymal stem cells toward the tendon lineage (Scx) at the expense of cartilage lineage (Sox9) via SMAD2/3 pathway activation [115]. Tendon-related markers, Scx, Col I, and Tenascin-c (Tnc) were induced by exposing human BMSCs, ASCs, and TCs to TGF-β3 in a two-step differentiation protocol [394]. However, despite this evidence confirming an early tendon differentiation, a downregulation in mRNA expression of decorin (Dcn), a protein with a crucial role in the collagen fibers maturation, has been documented in all cell types [394]. This led to the suggestion of a mutually inhibitory interaction between TGF-β family proteins and decorin during the process of collagen fiber maturation by demonstrating that an initial TGF-β3 dependent priming step (0–3 days) is necessary to induce in vitro TCs differentiation followed by a second step of TGF-β3- independent matrix deposition (3–10 days) [394,395]. A tendon-inductive in vitro role for TGF-β3 in combination with tendon-derived ECM (tECM) extracts has been further confirmed on human-derived mesenchymal stem cells (hASCs) plated on tissue culture or seeded on PCL-aligned scaffolds by recording increased Scx and Tnc expression as well as a greater production of Col I protein [206]. TGF-β3-mediated teno-inductive effects have also been verified with equine embryo-derived stem cells [396] and on hMSC and hBMNC cultured in fixed-length fibrin gels. The TGF-β3 hMSC and hBMNC-treated cells spontaneously synthesized narrow-diameter collagen fibrils and exhibited fibripositors (actin-rich, collagen fibril-containing plasma membrane protrusions) identical to those that occur during embryonic tendon development [397]. Even if the TGF-βs administration seems to be a promising inductive influence on in vitro tenogenesis, further in vivo studies for validation are required. Of note, TGF-β dysregulation has been related to tendinopathy and overuse (excessive physical activity) and is considered a major predisposing factor for the development of pathological condition [386]. In addition, many clinical trials seem to support the idea that prolonged TGF-β1 stimulation may be responsible for reduced ECM remodeling, leading to scar formation [384]. On the contrary, TGF-β3 administration to tenocytes minimized the extrinsic scarring, decreased tendon adhesion, and promoted tendon healing by downregulating the expression of Smad3 and upregulating the expression of Smad7 [398].

Beside the large availability of experimental research data on TGFβ roles in tenogenesis, further investigations are still necessary to better understand the complex mechanisms of TGF-β role and related pathways before considering their place in in vitro tendon-inductive protocols as well as to move toward their clinical application.

#### 2.5.3. BMPs

Bone morphogenic proteins (BMPs), BMP-12, -13, and -14 (also known as GDF-7, -6, and -5, respectively) are members of the TGF-β superfamily, and they have individually been shown to play important roles in chemotaxis, proliferation, matrix synthesis, and cell differentiation [167]. During tendon healing, BMPs are elevated at early stages and decrease gradually over time [399]. However, BMPs belong to the pleiotropic TGF-β superfamily and have diverse effects on cells. It has been reported that BMP-2 drives osteogenic differentiation, assigning it an important role in enthesis, meaning tendon to bone healing. BMP-2 was even able to induce new bone formation within the tendon, which is not desired in intratendinous healing [382]. However, Hoffmann et al. [400] demonstrated that the overexpression of constitutively active Smad8 molecules in mouse recombinant BMP2-expressing MSCs (C3H10T1/2-BMP2 cells) was able to suppress in vitro cellular development into osteo lineages and to induce tenogenic differentiation by enhancing Scx protein expression and simultaneously downregulating osteocalcin and other osteogenic-related markers [400]. These in vitro results, further supported by an in vivo Achilles tendon model, suggested that the Smad8 molecule inhibits the osteogenic pathway induced by BMP2 and promotes the tendon/ligament differentiation route [400]. The BMP-derived effect and relative mechanisms in modulating tenogenic differentiation were demonstrated in several preclinical settings [401,402] and reproduced in vitro on different stem cells sources. Furthermore, in vitro BMP-12 supplementation was able to promote equine BMSCs mesenchymal lineage differentiation by the induction of tendon-related markers including tenomodulin and decorin, as well as osteogenic ones including alkaline phosphatase and von Kossa staining [403]. BMP-12 treatment has been evaluated as teno-inductive on BMSCs and ADMSCs where the upregulation of tendon markers including Scx, Tnmd, Col I, Tnc, and Dcn, was recorded and confirmed in vivo (horse, dog, and rat) [382]. The BMP12 teno-inductive role was demonstrated in Rhesus BMSCs where transfection with BMP-12 was sufficient to induce differentiation into tenocytes by enhancing Col I and Scx, but not Col III mRNA expression [200]. Analogously, BMP-13 seems to be involved in promoting in vitro tenogenic differentiation on BMSCs incorporated in an engineered tendon matrix further confirmed by their effect on neotendon formation after implantation in an experimentally induced tendon injury model [404,405]. It was recently established that growth media supplemented with BMP-12, BMP-13 and ascorbic acid could induce pluripotent stem cells (hESC) to undergo in vitro tenogenic differentiation under low tension O_2_ (2% O_2_) through a stable transcription of tendon-linked and tissue specific gene upregulation combined with the deposition of a tendon-like matrix and elongated and cell-to-cell synapsing [167]. A forward step in hESC tenogenesis will facilitate the generation of enhanced in vitro studies. The teno-inductive influence of BMP-14 has also been evaluated mainly on tissue-derived progenitor cells (TSPCs). It has no effect on TSPCs proliferation but leads to a progressive loss in stemness by elevating the expression of Dcn, Scx and osteonectin, but reducing Tnc, Col I, and Col II [387]. However, the effect of BMP-14 seems to be highly stem cell-dependent. In multipotent adult adipose-derived rat MSCs, treatment with different concentrations of BMP-14 (0–1000 ng/mL) increased proliferation and induced more complete tenogenic differentiation with an upregulation of tenogenic gene markers (Scx, Tnmd, and Tnc) and tendon-specific (collagen type I, decorin, and aggrecan) markers codifying for ECM protein components [406]. Ciardulli et al. [196] reported that 100 ng/mL of BMP14 promoted a time-dependent expression of tenogenic markers (Col I, Col III, Dcn, Scx-a, Tnc, and Tnmd) by BMSCs and WJMSCs. Govoni et al. [202], studied the teno-inductive influence of hBMP-14 (also called GDF-5) in an alternate adult MSC source. In particular, hBMP-14 stimulation of hBMSC maintained on a synthetic three-dimensional (3D) microenvironment underwent an early commitment toward the tenogenic lineage as consequence of a combination biochemical and physical stimulation. In detail, under a multiphase 3D construct consisting of a braided hyaluronate elastic band merged with poly-lactic-co-glycolic acid growth factors (GFs)-loaded microcarriers, hBMP-14 was regularly delivered to hBMSCs stimulated with cyclic strain. The cooperative biochemical and physical stimuli induced a significantly increased expression of tenogenic markers, such as Col I and Col III, Dcn, Ssc, and Tnc after 3 days of dynamic culture [198]. The BMPs molecular signaling pathway involved in tendon differentiation is still unknown. BMPs can transduce the signal through the Smad pathway or through the mitogen-activated protein kinase MAPK pathway. Some research studied the BMPs pathway through changes in Smad-1/5/8 levels [407,408]; still, few studies have focused on the underlying molecular mechanism of tenogenesis. However, a recent study demonstrated that BMP-14 promoted tenogenic differentiation in hBMSCs by activating cytoskeleton reorganization signaling (stress fiber formation), e.g., keratin filament, activin A, cell adhesion, and extracellular matrix related signaling [409], demonstrating the involvement of alternative mechanisms of induction of in vitro tenogenic differentiation. In conclusion, despite the rigorous evidence collected to date on the in vitro teno-inductive role of BMP-12, -13, and -14 on the different typologies of tendon-related and adult MSCs, in vivo studies validating a physiological role and potential clinical impact of this GFs superfamily remain to be performed.

#### 2.5.4. CTGF

Connective tissue growth factor (CTGF) is involved in skeletal development and differentiation [410]. CTGF induced fibroblastic differentiation in hBMSC, increasing the expression of Col I and Tnc and reducing the capacity to undergo non-fibroblastic differentiation [384]. CTGF is able to induce mineralization in human periodontal ligament stem cells, but it is able to increase fibroblastic-related gene expression when combined with TGF-β1 [411]. Based on the in vitro evidence, CTGF seems to exert a dual differentiation effect toward fibroblastic or osteoblastic lineages modulated by the microenvironmental conditions [384].

More recently, CTGF was considered to be able to promote the in vitro tendon differentiation of mouse ASCs in a dose- and time-dependent manner. CTGF increased tendon-related genes and proteins such as Scx, Tnmd, and Col Ia. Its influence, mediated by MAPK kinase activation, induced ERK1/2 and FAK phosphorylation within 5 min and 15 min, respectively. The FAK/ERK1/2 signaling role in CTGF-induced tenogenesis was also demonstrated by inhibiting ASCs’ tenogenic differentiation and proliferation, blocking both the pathways by selective inhibitors SCH772984 and PF573228, respectively [412]. Of note, the use of antagonists has been associated with a parallel upregulation of chondrogenic (Acan) and osteogenic-related genes (Runx2). This teno-inductive CTGF-mediated pathway was confirmed in vivo with rat tendon CD146^+^ stem/progenitor cells by generating an siRNA knockdown of focal adhesion kinase (FAK) and extracellular signal-regulated kinases ERK1/2 [399]. Little information is available to date on the role of CTGF in tendon somatic cells. TSPCs exposed to CTGF and ascorbic acid in vitro increased ECM deposition by generating a cell sheet that can be used as an engineered tendon tissue for transplantation [214]. Furthermore, a CTGF transection construct was capable of inducing tenogenic differentiation of rat TSPCs in vitro, upregulating the expression of Scx, Tnmd, Col I, and Tnc. CTGF promoted its effect on TPSC via the Smad1/5/8 signaling pathway. The role of CTGF has also been confirmed in vivo. Knockdown of CTGF expression diminished tenocyte differentiation [413]. This study provided the evidence for the existence of a direct interaction between CTGF and BMP-12 involving the CR domain of CTGF. To date, it remains unclear how CTGF regulated tenogenic differentiation and whether its effect is linked to other GFs signaling and, in particular, to TGF-β family signaling pathways. Further investigation of CTGF and its potential crosstalk with TGF-β will likely provide an in-depth understanding of its roles in tendon regeneration [384].

#### 2.5.5. FGFs

Basic FGF (bFGF) is a member of the heparin-binding growth factor family and is known to be a potent stimulator of angiogenesis and cellular migration [382]. In vitro bFGF supplementation promoted the maintenance of differentiated cells despite the lower proliferation of hTSPCs in a TSPC–tenocyte co-culture [414]. Gonclavez et al. [389] reported that bFGF in combination with ascorbic acid exerted a teno-inductive in vitro effect on human amniotic fluid stem cells (hAFSCs) and adipose-derived stem cells hASCs. In both the cells, typologies increased the expression tendon-related markers (Scx, Tnc, Dcn, Col I a1, and Col III a1), even if hAFSCs appeared to be more responsive to bFGF stimulus [389]. Independently of stem cell source, the bFGF effect was strictly dose-dependent in vitro. Human BMSC exposed to low dosages (3 ng/mL) of FGF-2 displayed a biphasic response where initial proliferation was followed by differentiation through the upregulation of Col I, Col III, and Fnc. On the contrary, neither differentiation nor proliferation influence was induced by a high dose (30 ng/mL) of FGF-2 [415]. The tenogenic role of bFGF remains to be confirmed in vivo where contrasting evidence has been reported. Two in vivo studies using BMSCs lentiviral transfected with bFGF demonstrated the absence of any healing influence when cells were transplanted in a rat Achilles tendon defect model [416,417]. On the other hand, a direct bFGF administration through a hydrogel implantation apparently increased the ultimate strength and higher histological scores in a rat rotator cuff injury experimental model. This preclinical setting demonstrated that bFGF may have a role in driving tenogenic progenitor cells recruitment and activation by increasing at the healing sites the number of MSCs upregulating Scx [418]. On the contrary, FGF-4 use did not increase Scx expression in mouse limbs in both early and late developmental stages with no negative effects on Scx and ColIa1 gene expression in mouse C3H10T1/2 cells [384]. Similar conclusions were obtained by another study where a slight effect of FGF-4 on the differentiation of TSPCs isolated from the axial and limb in a series of developmental stages were recorded [419].

#### 2.5.6. IGF-1, VEGF, and PDGF

IGF-1 is involved in multiple processes in normal body growth and healing. It mediates all stages of wound healing, especially the inflammatory and the proliferative phases. IGF1 is known to play a critical role in the growth and adaptation of various musculoskeletal tissues (including skeletal muscle, bone, and cartilage), but less is known about tendons [382]. IGF-1 mainly seems to stimulate the proliferation and migration of fibroblasts and other cells at the injury site and to increase the production of collagens and other ECM components in these cells [420]. However, the in vivo evidence collected to date did not demonstrate any tenogenic role of IGF-1 in mediating the host–stem cells dialogue during healing. Indeed, adenoviral BMSCs transfected with IGF-1 did not enhance stem cell ability in stimulating transplanted tendon to recover its biomechanical property as well as to improve new ECM deposition [421]. However, the in vitro evidence remains limited and non-homogeneous in order to make any conclusion on the tenogenic role of IGF-1. For instance, Liu et al., reported that a combined treatment of IGF-1 and BMP-2 on rat TSPCs significantly increased adipogenic differentiation through a prostaglandin E (PGE) 2-mediated pathway [422] while Holladay et al. [387] reported that in vitro IGF-1 treatment alone promoted the proliferation and maintenance of TSC phenotypes, leading to a slight increase in Scx, no influence on Col I, and a downregulation of Col II expression [387]. The basal expression of the angiogenic factor, VEGF, is low in healthy tendon while it is reactivated during tendon healing [50,52,188] and upregulated in tendinopathy [48,423,424,425]. Despite VEGF having an effect on stromal cells, it is a highly specific growth factor for endothelial cells [426]. Analogously, it stimulates angiogenesis during tendon healing. Activation occurs early after tendon injury and persists during the whole inflammatory phase [50,52,188] followed by its reduction for modulating blood vessels organization during the proliferative and remodeling phases [427,428]. VEGF effects on in vitro tenogenic differentiation were evaluated on human tenocytes cultured with different concentrations of growth factor. VEGF was able to enhance tenocyte proliferation as well as to increase Scx and Col I expression by downregulating Col III [429]. More consistent are experiments performed to investigate the tenogenic in vitro influence of VEGF. However, most of these experiments have been performed using VEGF in combination with other growth factors [201,384], and the relative results will be discussed in the “Comparative Studies with Growth Factors” Section 2.5.7 below. Platelet-derived growth factor (PDGF-BB) is a potent mitogen for cells of mesenchymal origin, including fibroblasts, smooth muscle cells, and glial cells [430]. Several reports seem to confirm that PDGF plays an important role in tendon tissue homeostasis. Indeed, PDGF was essential to stimulate collagen, non-collagen protein production, and DNA synthesis in different types of rabbit tendons (intrasynovial intermediate and proximal segments of deep flexor tendons, and extrasynovial peroneus) during short-term explant cultures acting in a dose-dependent manner [431]. Different studies take advantage of PDGF supplementation for enhancing in vitro tenocyte activity, for inducing phenotype conversion of different stem cells toward tenocyte-tissue lineage, and for augmenting tendon tissue responses to biomaterials [432]. In this context, PDGF-BB supplementation or its incorporation in biomimetic scaffolds was able to stimulate the proliferation of rat tenocyte [433] and to induce tenogenic differentiation in ADSC [432].

#### 2.5.7. Comparative Studies with Growth Factors

The definition of an optimal protocol for in vitro tenogenic differentiation remains to be determined. Difficulties emerge in drawing together an efficient in vitro approach when we consider the range of different culture conditions, time points, and experimental setups proposed to date in the absence of meaningful and robust conclusions. In this context, the in vitro role of GFs is further complicated by the evidence that tenogenesis requires a combination of GFs with controlled concentrations and durations of exposure. However, some comparative experiments as well as in vitro trials of GFs co-supplementation merit consideration.

Comparative studies of GFs teno-inductive capacity seem to suggest a central role of TGFβ superfamily GFs members. hADSCs and hAFSC underwent comparative analysis of the in vitro influence of EGF, bFGF, PDGF-BB, and TGF-β1 [37]. The expression profiles of tendon-related genes (Col I, Col III, Dcn, Tnc, and Scx) revealed that TGF-β1 and EGF were the more efficient GFs in inducing an early (at day 7) upregulation of Scx (10 times over the other GFs) and Tnc, respectively. The influence of GFs changed in the later culture intervals. EGF controlled Col III expression at day 14, while TGF-β1 and PDGF-BB upregulated Col I (10 times more than the other growth) at day 21. Of note, the study demonstrated even that the expression profiles of tendon-related genes are clearly stem cell source-dependent, thus suggesting a different ability in human AFSCs and ADSCs in undertaking in vitro tenogenic lineage commitment [37]. Furthermore, TGF-β1 treatment was recently identified as a potent tenogenic phenotype convertor in rat BMSCs where its supplementation was able to promote the greatest upregulation of tenogenic-related genes and proteins in comparison to BMP-12, CTGF, and their combinations [226]. In addition, TGF-β1 in combination with BMPs appeared to be essential to preserve Scx-GFP expression levels in primary tenocytes after several days of culture. [282].

A further confirmation that TGFβ superfamily member (TGF-β3) may be early tendon inducer (inducing Scx overexpression) was demonstrated by testing the influence of different combinations of GFs (BMP-12, b-FGF, TGF-β3, CTGF, IGF-1) on human ADSCs, BMSCs, and TC during a two-step differentiation protocol in the presence of ascorbic acid [394]. Of note, a late inhibitory role of TGF-β3 was demonstrated through the downregulation of the tendon markers Dcn. On the contrary, BMP-12, b-FGF, and AA were active in inducing Dcn upregulation. Furthermore, the results confirmed a conserved role of TGF-β3 that was able to promote an early teno-inductive effect and a late inhibitory influence on collagen fiber maturation on all cell typologies. Moreover, BMP-12 as well as CTGF and IGF-1 seem to be subordinated to TGF-β3 in the induction of tendon-specific transcription factors with a late role in modulating the production of tendon-specific extracellular matrix molecules. TCs were the most responsive cell population to GFs stimulation. Although TC displayed basal values of Col I a1, Tnc, and Dcn expression similar to those recorded in ASCs and BMSCs, their response to a combined GFs stimulation was faster and of the greatest amplitude. TC primed with TGF-β3 showed an earlier overexpression of Scx and Mkx (first 3 days) followed by a marked upregulation of Dcn, Tnc, and Mkx [394]. The findings obtained in this study showed a crucial role for TGF-β3 as an inducer of tenogenic differentiation. However, its opposing effect in the late phase of tenogenesis as an inhibitor of fiber maturation suggests the need of a two-step protocol with the other GF to achieve effective tenogenic differentiation.

In another study, TGF-β3 and BMP12 tenogenic combinatory effects were demonstrated in equine ADSCs cultured in monolayer or in 3D on decellularized tendon matrix scaffolds preloaded with the GFs [434]. Mechanistic insights on TGFβ GF superfamily pathways were investigated by another comparative study where the tenogenic differentiation of mouse limb MSCs were induced using FGF-4, TGF-β3, TGF-β2, and PD18. A greater tendon-inductive influence was achieved by activating TGF-β2/3 signaling [115] that, additionally, played a role in suppressing cartilage marker Sox9 expression. However, no differences in the effects of TGF-β2 versus TGF-β3 nor synergetic effects were observed.

Gene response profiles of mice TSPCs seem to confirm that the tendon-inductive influence of TGF-β2 does not require any mechanical loading in addition to FGF-4. TGF-β2 upregulated Scx expression independently of developmental stages of origin, whereas mechanical loading affected late-stage TSPCs. When this former effect was compared for the two GFs, a persistent stimulatory tendon-inductive influence was confirmed for TGF-β2, whereas FGF-4 appeared to be anti-tenogenic [419].

In addition, the TGF-β tendon-inductive influence could require an inductive surrounding microenvironment. Indeed, while TGF-β3 or TGF-β3/BMP-12 upregulated Col IIa1, Col IIIa1, Tnc, Scx, and Mohawk in equine MSC in a monolayer culture, in 3D conditions (seeded on tendon matrix), they overexpressed Dcn and osteopontin by downregulating Smad8. Scaffolds preloaded with TGF-β3 or with TGF-β3/BMP12 promoted a tenocyte-like phenotype and improved cell alignment. This study showed that growth factor-induced tenogenic differentiation was also markedly altered by topographical constraints of decellularized tendon tissue by suggesting the idea that TGF-β3 may play a key role as mediator for tenogenic induction, while BMP-12 served as a modulator [434]. On the contrary, a tendon-inductive role for BMP-12 and BMP-12+IGF-1 has been proposed in a further study [435] carried out in equine BMCS supplemented alone or in combination with FGF-2, TGF-β1, IGF-1 (BMP-12+IGF-1, TGF-β1+IGF-1, and/or BMP-12+FGF-2). The concept of the authors was substantiated by BMP-12 increasing the expression of tendon-related genes Col III and Scx over the control cells at day 10 (cultured in 3D over a collagen hydrogel) compared to other GFs formulations (5, and 3-fold, respectively, over the levels induced by TGF-β1 and TGF-β1+IGF-1). Of note, BMP-12-induced Scx expression was significantly decreased by FGF-2 co-supplementation. In addition, BMP-12+IGF-1 significantly increased Col III expression over all groups (except BMP-12, BMP-12+FGF-2, and CTR) while BMP-12 and BMP-12+IGF-1 significantly stimulated Dcn [435].

The combined tenogenic differentiation action of the BMP-14, TGF-β3, and VEGF formulation was demonstrated on rabbit BMSCs [201]. In this study, the teno-inductive influence was evaluated in both 2D and 3D (fibrin-based constructs) cultures at 7 and 14 days by analyzing cell metabolism and collagen content, the gene expression of tenogenic markers, and the histological cell distribution and collagen deposition within 3D constructs. The results demonstrated that this formulation was the most effective in enhancing BMSC expression of Col Ia1, Col IIIa1, Tnc and Tnmd in both 2D and 3D cultures higher in BMSCS [201].

### 2.6. Co-Culture

**Figure 11:**
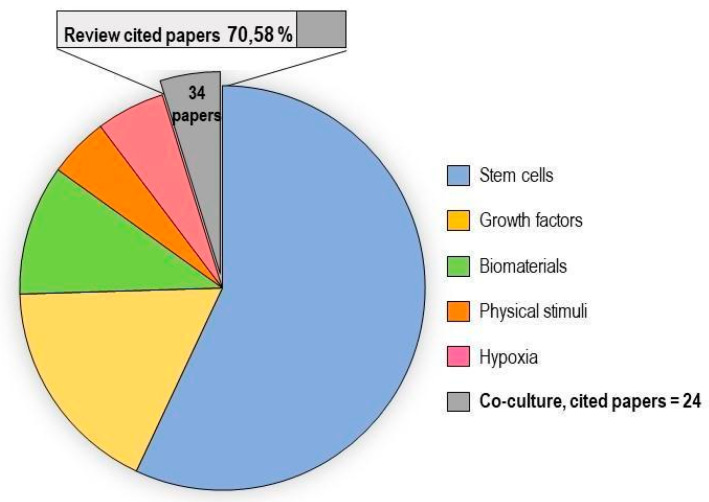
The gray section shows the percentage/number of papers available on the Scopus database obtained by combining key words in vitro stem cells tendon differentiation with co-culture. In the histogram, the relative percentage of cited papers is expressed as well as the relative number in the legend.

The co-culture technique has been widely used in tissue engineering of cartilage, bone, kidney, liver, lung, heart, and nerve to direct stem or progenitor cells differentiation [436]. The scientific production about co-culture in the field of in vitro tendon differentiation is still in development as the scientometric analysis has not yet revealed prominent available papers (Figure 11).

Co-culture aims at reproducing in vitro the molecular tissue microenvironment that stem cells may experience after transplantation. This cultural approach in theory may reproduce in vitro the complex stem cell host tissue paracrine dialogue undertaken during tissue development and regeneration by providing the tissue specific bioactive stimuli. The co-culture with fetal tissue may represent an in vitro system closer to that addressing tissue development, while the co-culture with TDSCs or adult tissues are probably more clearly referable to tissue repairing and regeneration.

From a methodological point of view, two types of co-culture may be performed: a direct and indirect incubation. Direct co-culture systems consist of two or more distinct cell types mixed and cultured together where cells interact with each other using the combination of paracrine, cell–ECM adhesion, and gap junction-mediated bioactive molecule signaling. In indirect co-culture systems, two or more distinct types of cells share the same environment without any physical contact. An indirect co-culture reproduces the environments of native tissue through the release of soluble factors in order to reproduce in vitro the dialogue between two different cell types [436].

Evidence in the literature showed the efficiency of co-culture techniques with fetal tendon explant with respect to fetal tenocytes and adult tissue or tenocytes. In fact, fetal explant co-culture may represent a good in vitro model to mimic tendon development. Fetal tendon is a very plastic tissue that during adulthood undergoes profound transformations. A study on sheep performed by Russo et al. [5] showed the modifications that occurred during tendon aging. For instance, fetal endotenon was more developed than in adult and cell phenotype shift during tendon maturation. In fact, in the fetal tendons, the cells were large with a rounded shape and were located on a layer of more compacted cells that expressed osteocalcin, VEGF, and nerve growth factor (NGF). During tendon development VEGF, NGF, blood vessels, and nerve fibers decreased. Moreover, contrary to adult specimens, cells in mid and late fetuses endotenon showed pluripotent stem cells markers. In adult tissue Col I, Col III, Scx B, Tnmd, Thbs4, and osteocalcin underwent dramatic reductions. In addition, TGF-β1 expression underwent a similar decrease [5].

According to this evidence, it can be supposed that signals from fetal tendons are different and more effective with respect to adult ones or tendon-derived stem cells alone. The hypothesis providing that fetal tendon explant can drive teno-differentiation more efficiently than fetal tenocyte or adult explant or tenocyte was demonstrated by Barboni et al. [184]. In particular, the transwell system of fetal explants and ovine Amniotic Epithelial Stem cells (oAECs) led these cells to differentiate toward the tenogenic lineage by a stepwise differentiation process. The exposition of oAECs to fetal tendon explants for 28 days results in a higher increase of tendon-related genes such as Scxb, Tnmd, Col I, Thbs4, increased protein Col I, and connexin 32 and 43, with respect to the oAECs co-cultured with adult tendon or tenocytes. Moreover, oAECs acquired a tenogenic phenotype as they organized themselves in 3D tendon-like structures [184]. Tendon differentiation occurred with a stepwise process confirmed by the fact that oAEC expressed mesenchymal markers after co-culture, such as α-SMA, which they did not possess when initially harvested [174,184,185,191,192].

The co-culture with tendon fetal explants is able to accelerate the tenogenic commitment of oAECs when seeded on a PLGA electrospun scaffold. In fact, oAEC on a high aligned PLGA scaffold displayed an early commitment toward tenocyte without any further stimuli, but in a co-culture system with fetal tendon, explant Tnmd and Col I gene expressions were significantly higher, as well as Col I protein deposition and orientation. Moreover, in this research, the stepwise differentiation process of oAEC was also evident through the upregulation of mesenchymal markers, such as Snail, Vimentin, and α-Sma and the downregulation of epithelial marker cytokeratin-8 [190].

Instead of tissue explants, sometimes, TDSCs are used in a co-culture system to induce tendon differentiation in stem cells, as they are tendon-derived stem cells and express tenogenic markers [21].

The choice to use TDSCs instead of the tissue explant as stimuli to drive stem cells’ tendon commitment may be due to the fact that native tissue is not often available, and a co-culture with cells could be more manageable than ones with tissue. Even if cell-to-cell interaction is different from tissue–cell interaction, a co-culture of TDSCs and stem cells could be useful to investigate the relationship between these two different cell types as a possible in vitro model to study tendon healing and regeneration.

Some researchers proved the positive outcome of co-culture between TDSCs and fetal or adult stem cells. The study of Muttini et al. [188] showed that oAECs are able to undergo tenogenic differentiation also in co-culture with tenocytes collected from adult equine tendons. The most interesting outcome was that the differentiation occurred infra-species (ovine/equine) as oAECs acquired tenogenic phenotype and genotype displaying Scx, Col I, and Col III gene expression as shown in Barboni et al. [184]. Pre-differentiated oAECs were also tested in vivo. After an in situ injection of differentiated oAECs in horses with acute tendon lesion, histological and immunohistochemical examinations in the explanted tendons demonstrated the low immunogenicity of oAECs as well as their regenerative potential in producing ovine collagen type I amongst the equine collagen fibers [188].

Li et al. [193] used both direct and indirect co-culture to induce tenogenic differentiation in human amnion-derived mesenchymal stem cells (hAMSCs). They cultivated hAMSCs with human anterior cruciate ligament fibroblasts (hACLFs) in a monolayer co-culture and in a transwell co-culture with and without growth factors stimulation (bFGF and TGF-β1). The final outcome demonstrated that hAMSCs in a transwell system stimulated with growth factors displayed a higher density of Col I, Col III, fibronectin, and Tnc, as well as mRNA expression of tenogenic markers Col I, Col III, fibronectin, and Tnc [193].

Wu et al. [437] demonstrated the advantage of the direct co-culture of rat TDSCs with BMSCs at a 1:1 ratio. At the end of the experiment, MSCs significantly upregulated tenogenic gene markers expression (Tnmd, Scx, Tnc, and Dcn), collagen matrix production, and enhanced also tendon injury healing in vivo [437]. Rat BMSCs cultured in a transwell system with tenocytes, proliferated after 3 days, and showed an upregulation of tendon/ligament-related genes Col I, Col III, Tnc, and Scx after 14 days of culture [438].

Schneider et al. [391] showed that canine MSCs in high-density direct co-culture with canine tenocytes are able to undergo tenogenic differentiation with a combination of growth factors IGF1 and TGFβ1 and through cultivation with the spent media from primary tenocytes. Immunoblotting and electron microscopy analyses on MSCs demonstrated the upregulation of Scx, Col I, Col III, Dcn, Tnmd, and b1-Integrin gene expressions as well as those of Shc and Erk1/2 belonging to the mitogen-activated protein kinase (MAPK) pathway [391].

Canseco et al. [439] experimented tendon differentiation in a direct co-culture system with pig MSCs and autologous cells derived from anterior cruciate ligament (ACL). MSCs and ACL were co-cultured in different ratios; however, significant results were detected in the 50/50 ratio. In fact, in this case, MSCs displayed the highest Col I and Tnc expression and the highest Col I/Col III ratio [440].

Additionally, the tenogenic potential of human ASCs were tested under a co-culture system. The direct co-culture of hTDSCs with hADMSCs at 1:3 ratios was reported to promote the expression of tenogenic genes such as Tnc and Scx [440].

An indirect co-culture system with tenocytes was able to induce the differentiation of hADMSCs into tendons-like cells increasing the expression of Scxb, Thsb4, and Tnmd genes and protein [441].

Human menstrual blood stromal stem cells (MenSCs) successfully differentiated after 3 weeks of indirect co-culture with Achilles tendon cells (ATCs) into tenogenic cells. This technique induced the production of the extracellular matrix and led to the expression of the specific Achilles tendon markers in MenSCs, such as Thsb4, Tnc, and Scx [442].

Both studies of Yu et al. [441] and Zheng et al. [442] above cited reproduced the physiological microenvironment not only with the co-culture system but also with the modulation of the oxygen tension. In fact, tendon is a poor vascularized tissue, and the oxygen consumption is lower than other body districts, so tenocytes possibly live in a very low oxygen environment [3,237]. In these experiments, co-culture was performed in normoxia (20% O_2_) and hypoxia (2% O_2_). The interesting common result in both conducted researches is that even if co-culture alone was able to commit stem cells toward tenogenic lineage, the combination with hypoxia was able to enhance the expression of tenogenic markers.

Co-culture with adult tendon explant may reproduce an environment mimicking tendon regeneration after injuries and can be used to study the crosstalk between stem cells and tissue. The advantage of using an ex vivo explant instead of tendon-derived cells is that the tissue preserves the cell’s native ECM niche, recreating a more realistic physiological environment in vitro fundamental to study the cells–ECM interaction [443].

Evidence showed that co-culture with adult tendon explant can drive mesenchymal stem cells toward tenogenic commitment. In particular, equine BMSCs in a transwell co-culture system with tendon tissue fragments expressed tendon-specific markers such as Dcn, Tnmd, Tnc, and Col I, and they also retained a tenocyte-like phenotype during monolayer culture [444].

Moreover, an indirect co-culture with human tendon explant and hASCs was used to obtain information about the bidirectional crosstalk between stem cells and the native tendon niche. The paracrine communication enhanced collagenolytic activity of MMPs in co-cultures at day 3, suggesting that ECM remodeling is triggered early in culture. Moreover, hASCs displayed the deposition of Col III and Tnc after 7 days and acquired more elongated structures in co-cultures [445].

The co-culture system can be used as a model to study the tendon-healing process and provide tendon differentiation also when tendon-derived stem cells or tendon explants are not provided.

An interesting study used the direct and indirect co-culture techniques as an in vitro model to understand the paracrine effect in the tendon-healing process. Lange-Consiglio et al. [186] investigated the immunomodulatory effect of equine amniotic membrane-derived MSCs (AMCs) both in direct and indirect co-culture systems with equine peripheral blood mononuclear cells (PBMCs). The results showed that AMCs inhibit the proliferation of PBMCs after allogeneic stimulation in both culture systems. They assumed that secreted factors released in the conditioned medium (CM) were responsible for the anti-proliferative effect, as no cell contact was required. Moreover, the injection of AMC-CM in spontaneous tendon injuries in horses showed no adverse effects such as fibrotic, metaplastic, or mineralization. In addition, the re-injury rate was lower in comparison with untreated cases after 2 years [186].

Wu et al. [446] provided an example of co-culture implied to induce tendon differentiation without tendon explants or derived cells. In particular, hADSCs co-cultured directly with hUVEC on aligned PLLA fibrous scaffolds with tendon differentiation medium, containing DMEM/F12 medium, 2% FBS, 20 ng/mL TGFβ3, plus endothelial growth medium, expressed higher tenogenic markers with respect to when cultured alone. In fact, Tnmd and Col I protein was robustly expressed, and the Tnc gene was upregulated [446].

Co-culture may represent a good in vitro model to understand the mechanisms involved in tendon differentiation, as it is able to provide a stepwise differentiation process when fetal tissues are used. Moreover, it could be a useful technique to replicate the first steps in tendon healing and to study the bidirectional communication occurring between stem cells, using tendon-derived stem cells and the native tendon niche with the implication of adult tissue.

## 3. Conclusions

In vitro teno-differentiation techniques represent a fundamental step prior to in vivo tendon disorder treatment with cell therapy or tissue engineering approaches. Tissue engineering refers to a multidisciplinary field that aims at inducing tissue repair or regeneration. Therefore, it involves the use of a combination of key factors, such as cells, scaffolds, biochemical inputs, and mechanical inputs to produce a functional tissue-like construct. Nowadays, a combination of two or more than one techniques seems to be the best way to induce tendon differentiation in stem cells [447].

Cells represent the building blocks of the engineered tissue. Undifferentiated, pre-differentiated, or differentiated stem cells can be used in tissue engineering. Several studies displayed the involvement of various types of stem cells (embryonic, fetal, and adult stem cells) from different sources with promising results. Multiple studies focused on a single cell type, but co-culture with fetal tendons could be a step forward, since it has been found to enhance differentiation, providing a stepwise differentiation process resulting in an increase in tendon specific markers [184]. Scaffolds supply mechanical stability and provide a 3D support for cell growth and differentiation. The electrospinning technique has been shown to be able to generate 3D scaffolds with highly organized nanofibers, similar to collagen fibers alignment in native tendon, improving the structural organization of the newly formed tissue-like construct during cell differentiation. Mechanical inputs with different loading features, provided by bioreactors, can dynamically affect the cell behavior within the scaffold, mimicking the physiological environment of the tendon. On the other hand, biologically active molecules (such as growth factors) or hypoxia can be used in synergy with the other factors to drive the process of cells maturation and differentiation.

Taken together, all these elements contribute to the formation of a tissue-engineered substitute to be used as an in vitro model or to be applied in tissue replacement techniques in vivo.

Several studies in the literature are focusing recently on a combined approach as a novel method for tendon tissue engineering, demonstrating how the cooperative effect of different factors improves the properties of the engineered tissue, compared to those obtained using a single factor. For instance, Testa et al. [448] cultivated C3H10T1/2 fibroblast cell line on a PEGylated–fibrinogen biomimetic matrix, exposing cells both to a biochemical stimulus, represented by TGF-β, and a mechanical input by applying uniaxial stretching. In vitro analyses demonstrated that the proposed combined approach led to a highly organized neo-extracellular matrix, with Col I fibers parallel to the stretching direction, reflecting the enhanced elastic modulus and endurance of the matrix [448]. Moreover, Rinoldi et al. [449] fabricated an electrospun nanofibrous 3D scaffold coated with a thin layer of mesenchymal stem cells-laden hydrogel. BMP-12 was added in the culture media, and the cell-laden scaffold was subjected to mechanical stimulation using a custom-built bioreactor. The cooperative effect of biochemical and mechanical stimulation showed enhanced cell viability, alignment, proliferation, and tenogenic differentiation [449]. The same approach was used by Govoni et al. [198] by fabricating a multiphase 3D construct composed of a hyaluronate elastic band merged with a fibrin hydrogel supplemented with human BMSCs and poly-lactic-co-glycolic acid microcarriers loaded with human GDF-5. The synergy between biochemical and mechanical inputs led to an increased expression of tenogenic markers, such as Col I, Col III, Dcn, Scx, and Tnc [198].

Collectively, we can conclude that in vitro techniques are fundamental to study tendon development, healing, and regeneration. Only with a validated and successful in vitro model will we will have a clear prospect of tendon biology and pathology in order to translate the knowledge in vivo to treat tendon disorders.

## Figures and Tables

**Table 1:** Ultrastructure and mechanical matrix properties in tendon tissue engineering.

Materials	Shape and Structure of the Scaffold	Mechanical Properties of the Scaffold	Ref.
Silk fibroin–collagen	As-spun scaffold → diameter = 1.15 μm; pore size = 43.79 μm^2^ Cross-linked scaffold with ethanol → diameter = 0.76 μm; pore size = 21.39 μm^2^ Cross-linked scaffold with methanol → diameter = 0.91 μm; pore size = 10.81 μm^2^	Cross-linked scaffold with ethanol → Stress = 1.2 MPa; Modulus of elasticity = 4.2 MPa Cross-linked scaffold with methanol → Stress = 2.92 MPa; Modulus of elasticity = 9.78 MPa	[324]
PLGA–Silk fibroin	Knitted silk scaffold (3 yarns, diameter = 10 μm) coated with electrospun PLGA nanofibers diameter = 200–700 nm Thickness of the knitted scaffolds = 0.6–1 mm Thickness of the coated hybrid scaffolds = 0.8–1.4 mm	Hybrid scaffold → failure load = 70.8 N; stiffness = 4.29 N/mm	[325]
CS–PCL	Random nanofibers = 215.79 nm Aligned nanofibers = 175.82 nm	n.d.	[328]
PEUUR	Four randomly oriented meshes with diameter: 0.28, 0.72, 0.82, 2.3 μm Two meshes with aligned fibers with diameter: 0.46 and 0.53 μm	n.d.	[335]
PEUUR	Aligned and randomly oriented fibers with different diameter size <1 μm, >1–2 μm<, and >2 μm	n.d.	[336]
PU	Aligned and random nanofibers with diameter of ≈657 nm; porosity = 85%; total pore area = 61.8 m^2^/g	Aligned nanofibers → Young’s modulus = 2500 kPa; Ultimate strength = 3520 kPa; Max strain = 1.08 Random nanofibers → Young’s modulus = 540 kPa; Ultimate strength = 1130 kPa; Max strain = 1.03	[337]
PLLA/PEO loaded with TSA	PLLA/PEO random mats → diameter = 1.51 μm PLLA/PEO/TSA random mats → diameter = 1.44 μm PLLA/PEO aligned mats → diameter = 1.73 μm PLLA/PEO/TSA aligned mats → diameter = 1.63 μm	PLLA/PEO/TSA random mats → Young’s modulus = 513.09 MPa; Tensile strength = 12.5 MPa PLLA/PEO/TSA aligned mats → Young’s Modulus = 51.59 MPa; Tensile strength = 2.70 MPa	[338]
PLGA (85:15)/COL/PU	Randomly oriented scaffolds with average fiber diameter size of 1.92 μm and pore size of 174.7 μm^2^ Aligned fiber scaffold with average fiber diameter size of 0.712 μm and pore size of 6.75 μm^2^	Random fiber 1.92 μm → Young’s Modulus = 20.76 MPa; Ultimate Tensile Strain = 56.16%; Tensile Stress = 1.17 MPa Aligned fiber 0.712 μm → Young’s Modulus = 38.11 MPa; Ultimate Tensile Strain = 82.99%; Tensile Stress = 1.80 MPa	[350]
PLGA (50:50)/COL1/PU	Randomly oriented scaffolds with average fiber diameter size of 2.82 μm and pore size of 211.3 μm^2^ Aligned fiber scaffold with average fiber diameter size of 0.759 μm and 4.67 μm^2^	Random fiber 2.82 μm → Young’s Modulus = 75.4 MPa; Ultimate Tensile Strain = 28.55%; Tensile Stress = 0.86 MPa Aligned fiber 0.759 μm → Young’s Modulus = 52.46 MPa; Ultimate Tensile Strain = 79.48%; Tensile Stress = 2.46 MPa	
PLGA (85:15)	Aligned fleeces → diameter = 2.5 μm Random fleeces → diameter = 2.1 μm	Aligned fibers → stress = 26.02 MPa and strain = 344% Random fibers → stress = 15 MPa and strain = 240%	[190]
PCL and PLLA	Sheets of aligned PCL → diameter = 898 nm; pore size = 14.3 μm Sheet of aligned PLLA → diameter = 869 μm; pore size = 21 μm	Braided PCL scaffolds → Young’s Modulus = 45.96 MPa; Ultimate stress = 19.99 MPa; Ultimate Strain = 0.62 mm/mm; Stiffness = 11.25 N/mm; Stacked PCL scaffolds → Young’s Modulus = 66.48 MPa; Ultimate stress = 8.73 MPa; Ultimate Strain = 0.24 mm/mm; Stiffness = 36.51 N/mm; Braided PLLA scaffolds → Young’s Modulus = 45.57 MPa; Ultimate stress = 5.74 MPa; Ultimate Strain = 0.50 mm/mm; Stiffness = 5.94 N/mm; Stacked PLLA scaffolds → Young’s Modulus = 118.47 MPa; Ultimate stress = 5.76 MPa; Ultimate Strain = 0.22 mm/mm; Stiffness = 24.31 N/mm;	[367]
PCL	Random multilayer scaffolds → diameter = 1.76 μm; thickness = 0.75 mm Aligned multilayer scaffolds → diameter = 1.57 μm; thickness = 0.43 mm	Random multilayer → Elastic Modulus = 12 MPa; Yield Strength = 0.5 MPa Aligned multilayer → Elastic Modulus = 18 MPa; Yield Strength = 1.5 MPa	[368]
PLLA	Mat thickness ranged between 0.14 and 0.17 mm Aligned fibers = 430 nm Random fibers = 450 nm	Aligned fibers → Stiffness = 3.48 N/mm; Failure force = 1.88 N; Young’s Modulus = 22.76 MPa Random fibers → Stiffness = 0.07 N/mm; Failure force = 0.17 N; Young’s Modulus = 0.63 MPa	[369]
PLGA (85:15)	Aligned scaffold →thickness = 0.22 mm; diameter = 615 nm; pore diameter = 4.228 μm; porosity = 80.745%; permeability = 7.87 × 10^−12^ m^4^/N s Random scaffold → thickness = 0.19 mm; diameter = 568 nm; pore diameter = 4.914 μm; porosity = 81.760%; permeability = 5.72 × 10^−12^ m^4^/N s	Aligned scaffolds → Elastic Modulus = 341 MPa; Yield Strength = 9.8 MPa; Ultimate Stress = 12 MPa and Strain = 8% Random scaffolds → Elastic Modulus = 107 MPa; Yield Strength = 2.5 MPa; Ultimate Stress = 3.7 MPa and Strain = 80%.	[370]
PLLA/COL1 (75:25)	Individual aligned fiber with diameter = 0.36 μm and bundle diameter = 624.9 μm	Bundle PLLA/Col1 (75:25) → failure stress = 11.3 MPa; work to failure = 0.225 J/mm^3^;	[371]
PLLA/COL1 (50:50)	Individual aligned fiber with diameter = 0.39 μm and bundle diameter = 643.1 μm	Bundle PLLA/Col1 (50::50) → failure stress = 6 MPa; work to failure = 0.208 J/mm^3^;	
PCL	PCL yarns with diameter = 208.5 μm Yarn diameter size = 460.2 nm, pore size = 12.2 μm Random PCL diameter = 484.5 nm, pore size = 2.1 μm Aligned PCL diameter = 452.3 nm, pore size = 1.4 μm	Yarn PCL woven fabrics → Young’s modulus = 70 MPa; Ultimate tensile strength = 10.7 MPa; Elongation at failure = 48% Random mesh → Young’s modulus = 5.2 MPa; Ultimate tensile strength = 2.2 MPa; Elongation at failure = 240% Aligned mesh → Young’s modulus = 13.6 MPa; Ultimate tensile strength = 5 MPa; Elongation at failure = 45%	[373]
PCL/DT-NPs	PCL/DT-NP scaffold PCL and PCL/DT-NP twisted yarn using 12 threads Diameter range 313–346 μm	PCL → Young’s modulus = 12 MPa; Strain at break = 3.4 mm·mm^−1^; Stress = 2.9 MPa PCL/DT-NP2.5 → Young’s modulus = 18 MPa; Strain at break = 3.9 mm·mm^−1^; Stress = 4.2 MPa PCL/DT-NP5 → Young’s modulus = 22 MPa; Strain at break = 4.2 mm·mm^−1^; Stress = 4.8 MPa	[304]
PLLA/PCL	Braided aligned PLLA and PCL nanofibers with diameter ≈990 and 945 nm, respectively. Varying number of stitches (SPI) (8, 12, 16, 20 and 24) →↑ braid angle from 47° to 67°	PCL/PLLA (100/0) → Ultimate strength = 50.57 MPa; ultimate strain = 1.01 mm/mm; Young’s modulus = 121.21 MPa PCL/PLLA (75/25) → Ultimate strength = 20.30 MPa; ultimate strain = 2.30 mm/mm; Young’s modulus = 78.71 MPa PCL/PLLA (50/50) → Ultimate strength = 4.64 MPa; ultimate strain = 0.75 mm/mm; Young’s modulus = 14.79 MPa PCL/PLLA (25/75) → Ultimate strength = 2.78 MPa; ultimate strain = 1.85 mm/mm; Young’s modulus = 5.25 MPa PCL/PLLA (0/100) → Ultimate strength = 13.47 MPa; ultimate strain = 0.75 mm/mm; Young’s modulus = 48.50 MPa Different braiding angles →↑ strength, Young’s Modulus and yield strength and ↓ yield strain with the braiding angles of 67° (8 SPI) compared to 47° (24 SPI)	[374]
PLGA (85:15)	Aligned fibers with diameters 320 nm, 680 nm, and 1.80 μm	The UTS and yield strength of the scaffolds remained unchanged whether is the fiber diameter size. The tensile modulus increased by increasing fiber diameter size while elongation at break and ductility decreased when the fiber diameter size in increased.	[375]
PCL	Four PCL mats with randomly oriented fiber and different diameter size; 0.11, 0.78, 1.88, and 3.43 μm	fiber diameters (range of 0.1–3.4 μm) → Young’s modulus = 7.6–30.6 MPa; ultimate tensile strength = 0.9–6.3 MPa; strain at break = 49–442%	[376]
PLGA (85:15)	Aligned nanofibers → diameter = 615 nm; pore size = 4.23 μm; porosity = 80.75% Random nanofibers → diameter = 667 nm; pore size = 4.91 μm; porosity = 81.76%	n.d.	[377]
PLGA (85:15)	Meshes of randomly oriented fiber with diameter nano-1 (390 nm), nano-2 (740 nm), micro (1.42 μm) Meshes of aligned fibers with diameter nano-2 (740 nm)	n.d.	[378]

polylacticacids (PLLA); polycaprolactones (PCL); poly (lactic-co-glycolic) acids (PLGA); poly(esterurethane) urea (PEUUR); polyurethane (PU); polyethyleneoxide (PEO); chitosan (CS); iron oxide nanoparticles (DT-NP); collagen type 1 (Col I). not determined (n.d.).

**Table 2:** In vitro assessment of teno-inductive properties of scaffolds on stem cells.

Cells	Mechanical Properties of the Bio-Hybrid In Vitro	Major In-Vitro Outcome	Ref.
BMSCs	After 21 days of culture Acellular scaffold → failure load = 61.5 N; stiffness = 5.92 N/mm Bio-hybrid scaffolds FGF (−) → failure load = 68.2 N; stiffness = 5.53 N/mm Bio-hybrid scaffolds FGF (+) → failure load = 82.7 N; stiffness = 6.97 N/mm	Bio-hybrid scaffolds (FGF +/−) → viable cells on the surface and in the depths of the scaffolds with higher viability on scaffolds FGF + ↑ mRNA expression Col I, Col III, fibronectin and biglycan and collagen ECM content at day 14 and 21, respectively, in scaffold FGF (+) respect to FGF (−).	[325]
Human primary BMSCs	n.d.	Aligned/random scaffolds: No difference in cell adhesion, and fibroblast morphology was observed onto both scaffold Cells aligned parallel to the direction of the nanofiber orientation. No difference has been detected for *Tnmd* gene expression on the aligned/random scaffold while *Col I* and *Col III* was upregulated on aligned scaffold.	[328]
Rat BMSCs	n.d.	Cells acquired spindle-like morphology on aligned fibers respect to random ones. ↓ cellular density by increasing fiber diameter size. ↑ cellular aspect ratio by increasing fiber diameter size and alignment ↓ mRNA *ColI a1*, *Tnmd* and decorin by increasing fiber diameter and alignment while ↑ mRNA *Scx* by increasing fiber diameter and decreasing fiber alignment	[335]
Multipotent fibroblastic C3H10T1/2 cells	n.d.	↓ cell density and ↓ mRNA decorin and *Col I* gene expression by increasing fiber diameter size after 7 days culture while ↑ mRNA *Col I* gene expression by decreasing fiber diameter after 14 days culture	[336]
Human LF	n.d.	Aligned/random nanofibers → no difference in cell proliferation and adhesion while ↑ collagen content in aligned nanofibers respect to random ones. By applying 5% uniaxial strain for 24 h at a frequency of 12 cycles/min → no histological difference between aligned nanofibers under static and dynamic conditions, ↑ collagen ECM in the aligned nanofibers under dynamic conditions. Random nanofibers → cells acquired a spindle-like morphology under only dynamic conditions.	[337]
Mouse tail TSPCs	n.d.	Aligned/random (+/− TSA) →↑ cell elongation and ↓ nuclear shape Aligned/random (+/− TSA) → no differences in cell proliferation and adhesion Aligned-TSA →↑ tenogenesis protein (Col I, Col V, Tnmd and Epha4), ↑ ScxGFP protein expression and mRNA expression of *Scx*, *Mkx*, *Eya1*, *Eya2*, *Six2*, *HoxA11* and *Egr1*, and ↑ HADC 3 and 4 and ↓ HDAC 1 compared to other groups,	[338]
Human iPSCs from HFF (human foreskin fibroblast)	n.d.	Aligned/random nanofibers: no differences in cell proliferation rate and adhesion. Cells seeded onto aligned nanofibers present a fibroblastic phenotype while those onto random nanofibers show a stellate-patterned morphology. Aligned nanofibers: ↑ mRNA of tendon-related genes (*Scx*, *Mkx*, *Tnmd*, *HoxA11*, *Epha4*, *Col Ia1*) and mRNA of integrin a1, a2, a5, b1 and myosin II B.	[340]
AECs	n.d.	Aligned/random →↓ DNA quantity and cell proliferation Aligned/random →↑ mRNA *Snail* and *Vimentin*, and ↑ *α*-SMA and ↓ Cytokeratin-8 protein expression Aligned/random →↑ mRNA *Tnmd* and *Col I* after 48 h culture.	[190]
Human BMSCs	n.d.	Cells were homogenously distributed and showed an elongated morphology on the stacked scaffold compared to the braided ones. Braided scaffolds: ↓ cell infiltration and ↓ cell distribution homogeneity. Stacked scaffolds: ↑ cell proliferation and Col I ECM deposition with an enhanced deposition in the case of PLLA scaffolds. Both braided and stacked PCL and PLLA scaffolds upregulated the expression of *Scx* with a strong enhancement on braided PLLA scaffolds at day 7 of culture. Braided and stacked scaffolds: No differences were seen in the expression of tenogenic transcription factor Mkx and ECM glycoprotein Tnc. Stacked scaffolds downregulate the expression of Col Ia1 and Col IIIa1 compared to the braided ones.	[367]
Human ADSCs	Aligned scaffolds showed significant increase in Young’s modulus and yield stress along the axis of fiber alignment compared to random one after 28 days of culture.	Aligned/random scaffolds → no difference in cell proliferation, GAGs and Col I and Col III content. Both scaffolds expressed tendon related genes markers with ↑ mRNA *Tnmd* and Col3A1 in aligned scaffolds.	[368]
Human tendon Progenitor Stem Cells	n.d.	Aligned fibers →↑ tenogenic markers scleraxis, eya2, *Col I*, *Col III*, *Col XIV*, elastin, integrin α1, α5, β1 and myosin II. Random fibers: ↑ mRNA *Ocn* and *Alp* gene expression compared to aligned fibers.	[369]
Human rotator cuff fibroblast-like cells	Aligned cellular → Elastic modulus = 350 MPa; Ultimate stress = 6 MPa; Yield strength = 6 MPa Random cellular → Elastic modulus = 120 MPa; Ultimate stress = 1 MPa; Yield strength = 1 MPa	Aligned/random scaffolds: no difference in cell proliferation and adhesion. Cells acquired an elongated shape on the aligned scaffolds while maintaining their polygonal shape on the random ones. Aligned scaffolds: ↑ mRNA integrin *α2* and *Col I* while similar mRNA gene expression for α5, β1 and *Col III* respect to random fibers. Similar matrix deposition in terms of *Col I* and *Col III* was seen with oriented collagen matrix along the aligned fiber.	[370]
Human tenocytes and human ADSCs	n.d.	Human tenocytes → cells elongated along the aligned fibers. All scaffolds types (random, aligned woven fabrics) expressed Tnmd and Col I. ↑ mRNA tendon-related genes (*Tnc*, *Col III*, *Col II* and *Tnmd*) in woven fabrics compared to aligned and random groups. Human ADSCs →↑ proliferation rate, cell infiltration, and ↑ mRNA of *Scx*, *Col I*, *Tnmd* gene expression on woven fabrics compared to aligned and random groups. Co-culture/tri-culture →↑ mRNA expression *Scx*, *Tnc*, *Tnmd*, *A-VEGFA* and *ANGPT2* in the tri-culture system compared to other groups Dynamic culture by applying a 4% strain at frequency of 0.5 Hz for 2 h per day Dynamic/static culture →↑ Tnmd and Col protein and ↑ tendon related gene expression under dynamic stretch with tri-culture system compared to static condition.	[373]
Human ADSCs	n.d.	Aligned PCL/DT-NP5 yarns under static and magnetic stimulation conditions → no differences in cell activity while ↑ in cell alignment and elongation along the longitudinal direction of the fibers under magnetic stimulation. ↑ mRNA *Dcn*, *Col Ia1*, *Col IIIa1*, *Tnc* under both conditions while ↑ mRNA *Tnmd* and *Scx* and ↓ mRNA osteogenic marker (*RUNX2*) only under magnetic stimulation.	[304]
Human iPSC-MSCs	n.d.	Three days under static condition then for 7 days under 3% strain at 0.25 Hz for 2 h/day. PLLA/PCL scaffolds →↑ cell adhesion in PLLA compared to PCL. Cells were more elongated on PLLA scaffolds with 8 SPI compared to other groups (PCL 8 SPI, PLLA, and PCL 24 SPI). PLLA/PCL scaffolds → no difference in *Scx* and *Tnmd* mRNA expression while ↑ *Col I*, *Col III*, *RUNX2*, *Ocn* mRNA expressions and ↑ in Col I and Tnmd protein expressions in PLLA respect to PCL. Static/dynamic conditions →↓ *RUNX2* and *Ocn* mRNA expression under cyclic condition compared to static one. Different braiding angles → cells showed elongated morphology on PLLA with 8 SPI while those seeded on PLLA with 24 SPI showed a rounder morphology. No difference in *SCX* and *TNMD* between PLLA and PCL with 8 and 24 stitches, ↓ *Col I*, *Col III*, *RUNX2*, and *Ocn* mRNA expression on PLLA with 8 SPI compared to 24 SPI after 10 days culture under cyclic condition.	[374]
Human rotator cuff fibroblast	n.d.	The cells were more aligned and elongated in the fibers with larger fiber diameter size. Smaller fiber diameter size →↑ cell proliferation and ↑ in collagen and proteoglycans synthesis. Larger fiber diameter size →↑ mRNA expression of *Col I*, *Col III*, *Col V* and *Tnmd*.	[375]
Human MSCs	n.d.	1% strain at 1 Hz for 90 min twice a day Aligned/random scaffolds →↑ cell proliferation on random scaffolds after 28 days of dynamic culture with compared to other groups. Aligned/random → cells acquired an elongated morphology on aligned scaffolds (static and dynamic conditions) and on random scaffolds under dynamic conditions while those on random scaffolds remained cuboidal. Static/dynamic culture →↑ Col I ECM content in aligned (static) and random (dynamic) scaffolds while ↑ Col I and Col III ECM content on only aligned (dynamic) Aligned scaffolds under static/dynamic culture → no change in Col I and *Tnmd* mRNA expression between groups; ↑ *Col III*, fibronectin, and Tenascin-C and ↓ *Scx* mRNA expression under dynamic culture compared to static one after 28 days culture. ↑ integrin α2, α5, β1 expression on aligned scaffolds under dynamic condition.	[377]
Human rotator fibroblast	n.d.	Nano-/micro-fibers: ↑ cell adhesion, spreading and elongation by ↑ fiber diameter size. No differences in cell viability and proliferation. Nano-/micro-fibers: ↑ collagen content in nano-1 and nano-2 compared to micro scaffolds while ↑ *Col I* and *Col III* in the micron scaffold compared to the nano ones. ↓ mRNA expression *α2* and ↑ mRNA expression *β1* and ↑ mRNA expression *RhoA* and *Rac1* on the nanofibers compared to microfibers after 7 days culture Aligned/random fibers: ↑ cell adhesion and alignment and ↓ cell proliferation onto aligned fibers compared to random ones.	[378]

Bone-marrow derived Mesenchymal Stem Cells (BMSC); Amniotic Epithelial Stem Cells (AEC); Mesenchymal Stem Cells (MSC); Adipose-Derived Stem Cells (ADSC); induced Pluripotent Stem Cells–Mesenchymal Stem Cells (iPSC–MSC); Tendon Stem/Progenitor Cells (TSPC); Ligament fibroblast (LF). not determined (n.d.).

**Table 3:** In vivo performance of bio-hybrids constructs in tendon tissue engineering (↑ = increase; ↓ = decrease).

Animal Model, Tissue Site, and Duration of Implantation	Mechanical Properties of the Scaffold Following Implantation	Biological Outcomes	Ref.
Rat Achilles Tendon model, 2 and 4 weeks	Aligned-TSA vs. Aligned vs. Random-TSA vs. Random Stiffness = 29 vs. 25 vs. 20 vs. 19 MPa Failure Force = 38 vs. 32 vs. 32 vs. 31 N Stress at Failure = 6.6 vs. 5 vs. 5.5 vs. 4.2 MPa Young’s Modulus = 51 vs. 33 vs. 31 vs. 32 MPa	Aligned fibers: regulation of typical tendon structure with cell alignment along the fibrous axis ↑ matrix deposition and ↑ mRNA *Mkx*, *Tnmd*, *bgn*, *Fmod*, *Col I*, *Col III*, and *Dcn* on aligned fibers especially aligned-TSA vs. other groups ↑ collagen fibril diameter in aligned-TSA vs. other groups	[336]
Rat Achilles Tendon model, 2 and 4 weeks	Aligned vs. random Stiffness = 32.08 vs. 20.95 N/mm Failure Force = 50.47 vs. 42.85 N Stress at Failure = 5.91 vs. 4.90 MPa Young’s Modulus = 20.24 vs. 14.40 MPa	Aligned nanofiber: ↑ mRNA of tendon-related ECM gene markers *Col Ia1*, *Col Va1* and *Bgn*, *Scx*, *HoxA11*, *Tnmd* and *Fmod* and ↓ mRNA of *Ocn* and *RUNX2*. ↑ deposition of ECM (*Col I* and *Dcn*) after 4 weeks implantation.	[340]
Mouse skeletal muscle, 1 and 6 weeks Mouse skin, 1 week	n.d.	Cytotoxicity model: linear cell distribution with an elongated morphology and aligned collagen bundles formation on the aligned fibers Aligned fibers: ↑ collagen I ECM deposition with aligned structure.	[369]

not determined (n.d.).
